# A Bayesian multi‐arm multi‐stage clinical trial design incorporating information about treatment ordering

**DOI:** 10.1002/sim.9752

**Published:** 2023-05-09

**Authors:** Alessandra Serra, Pavel Mozgunov, Thomas Jaki

**Affiliations:** ^1^ MRC Biostatistics Unit University of Cambridge Cambridge UK; ^2^ Faculty for Informatics and Data Science University of Regensburg Regensburg Germany

**Keywords:** adaptive designs, Bayesian inference, infectious diseases, multi‐arm multi‐stage, order restriction

## Abstract

Multi‐Arm Multi‐Stage (MAMS) designs can notably improve efficiency in later stages of drug development, but they can be suboptimal when an order in the effects of the arms can be assumed. In this work, we propose a Bayesian multi‐arm multi‐stage trial design that selects all promising treatments with high probability and can efficiently incorporate information about the order in the treatment effects as well as incorporate prior knowledge on the treatments. A distinguishing feature of the proposed design is that it allows taking into account the uncertainty of the treatment effect order assumption and does not assume any parametric arm‐response model. The design can provide control of the family‐wise error rate under specific values of the control mean and we illustrate its operating characteristics in a study of symptomatic asthma. Via simulations, we compare the novel Bayesian design with frequentist multi‐arm multi‐stage designs and a frequentist order restricted design that does not account for the order uncertainty and demonstrate the gains in the sample sizes the proposed design can provide. We also find that the proposed design is robust to violations of the assumptions on the order.

**Abbreviations**
FWER: family‐wise error rate
MAMS: multi‐arm multi‐stage
ORD: order restricted design

## 1 INTRODUCTION

The development process of clinical trials can be lengthy and costly.^1^ Adaptive clinical trial designs, which allow multiple treatment arms to be tested simultaneously,^2^, ^3^, ^4^ can efficiently improve this process by allowing unpromising treatments to be dropped during the trial or by stopping the trial early for overwhelming benefit at a series of interim analyses.

Serra et al^5^ have recently proposed an order restricted design that extends the multi‐arm multi‐stage framework^6^, ^7^, ^8^ when an “order” (ie, a monotonic relationship) among the treatment effects can be assumed. Such an order can occur naturally, for example, when multiple doses or administration schedules of the same treatment are tested, when nested combinations of treatments are investigated or when different treatment durations are simultaneously compared against a standard regimen. In their work, Serra et al^5^ assumed that there was no uncertainty about the order of the treatment effects. While this assumption is highly plausible in some settings, there could be uncertainty, for example, when an increase in side effects or compliance with the treatment (eg, for longer treatment durations) lead to reduced effects on the treatment efficacy. Thus, methodology that is able to relax the assumption on the order of the treatment effects is needed for such settings.

Although MAMS designs are commonly implemented using frequentist approaches, they can be extended to the Bayesian framework.^9^, ^10^ Bayesian sequential designs have been proposed by several researchers for clinical trials.^11^, ^12^, ^13^, ^14^, ^15^, ^16^ These designs are more flexible compared to frequentist designs as they allow incorporation of prior information about the efficacy of the treatment. The frequentist operating characteristics of Bayesian designs are important,^17^, ^18^, ^19^ especially in confirmatory or late phase trials where it is required to control the risk of false positive results, which is the erroneous conclusion that new experimental treatments are efficacious. There are both proposals of Bayesian designs that do not consider control of the type I error^11^ and that do (e.g. Ryan et al^15^, ^16^). In the Bayesian setting, the posterior probabilities can be used to derive stopping boundaries, which are used to test the hypotheses and can be constructed to provide control of the frequentist type I error rate at the desirable level α.

In this work, we propose a Bayesian multi‐arm multi‐stage (B‐MAMS) design that incorporates the information of the order among the treatment effects building on the frequentist framework developed by Magirr et al.^3^ The proposed design can provide control of the overall type I error under specific values of the control mean and does not assume any parametric arm‐response model. The objective of the design is to identify all promising arms (eg, treatment durations, doses or combination of treatments), including the one associated with the smallest relevant treatment effect.

The rest of the manuscript continues as follows. A case study is introduced in Section 2 before a detailed description of the design is provided in Section 3. Section 4 revisits the case study through an extensive simulation study before a discussion is provided in Section 5.

## 2 CASE STUDY SETTING

The *Tiotropium add‐on therapy in adolescents with moderate asthma: A 1‐year randomized controlled trial* [NCT01257230]^20^ is a Phase III study assessing the efficacy and safety of 5μg or 2.5μg once‐daily tiotropium via Respimat added to inhaled corticosteroid (ICS) with or without a leukotriene receptor antagonist in adolescent patients with moderate symptomatic asthma. The primary efficacy outcome was change from baseline in peak ${\text{FEV}}_{1}$ within 3 hours after dosing (peak FEV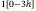) measured after 24 weeks of treatment. Serra et al^5^ revisited this study proposing an Order Restricted Design (ORD) when a monotonic dose‐response relationship could be assumed. However, their design effectively disregarded the historical information coming from a previous study called the *Tiotropium in asthmatic adolescents symptomatic despite inhaled corticosteroids: A randomised dose‐ranging study* [NCT01122680].^21^ This Phase II study evaluated the safety and efficacy of three doses—5μg,2.5μg,1.25μg—of once‐daily tiotropium versus placebo in three 4‐week treatment periods in adolescents with asthma. The primary efficacy outcome was change from baseline in peak ${\text{FEV}}_{1}$ within 3 hours after dosing (peak FEV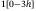).

In the following section, a Bayesian design which allows incorporation of the information from the Phase II NCT01122680 study^21^ for the design and analysis of the Phase III trial^20^ will be proposed. Moreover, the proposed Bayesian design will be able to account for the uncertainty in the monotonic dose‐response relationship.

## 3 BAYESIAN ORDER RESTRICTED DESIGN

Consider a clinical trial setting with K−1 active treatment arms, $T_{1},\ldots ,T_{K-1}$, a control arm, $T_{0}$, and J stages at which treatment arms can be dropped or the trial could be stopped for efficacy or futility. Let $t_{j}$ be the proportion of the total sample size at which the interim analysis j is done. The patients' responses are assumed to be independent and normally distributed at each stage j with known variances $\sigma_{k}^{2}=\tau^{-1},k\in \{0\;\text{(control arm)},1,\ldots ,K-1\}$. Let $Y_{i}^{\left(k\right)}∼N(\mu^{\left(k\right)},\sigma_{k}^{2}),k\in \{0,\ldots ,K-1\},i=1:n_{j}^{\left(k\right)},j\in \{1,\ldots ,J\}$ be the observation of the ith patient on treatment k and $n_{j}^{\left(k\right)}$ be the number of patients on arm k up to stage j. Define $n_{j}=(n_{j}^{\left(1\right)},\ldots ,n_{j}^{\left(K-1\right)})$ as the vector of sample sizes on the active arms at stage j and $n_{j}^{\left(0\right)}$ as the sample size on the control arm at stage j. Let ${\bar{Y}}_{j}^{\left(k\right)}={\left(n_{j}^{\left(k\right)}\right)}^{-1}\sum_{i=1}^{n_{j}^{\left(k\right)}}Y_{i}^{\left(k\right)},\;k\in \{1,\ldots ,K-1\}$ be the cumulative outcome mean on treatment k up to stage j and the vector of outcome means up to stage j is ${\bar{Y}}_{j}=({\bar{Y}}_{j}^{\left(1\right)},\ldots ,{\bar{Y}}_{j}^{\left(K-1\right)})$. Denote the covariance matrix of the joint distribution of the active treatment responses by ∑, which corresponds to a diagonal matrix with the vector $\left(\sigma_{1}^{2},\ldots ,\sigma_{K-1}^{2}\right)$ on the diagonal and zero on the off‐diagonal.

Let $\theta^{\left(k\right)}=\mu^{\left(k\right)}-\mu^{\left(0\right)}$ be the treatment effect of arm k∈{1,…,K−1} compared to control and $\theta =(\theta^{\left(1\right)},\ldots ,\theta^{\left(K-1\right)})$ be the vector of treatment effects. The null hypotheses of interest are $H_{01}:\{\theta^{\left(1\right)}\leq 0\},\ldots ,\;H_{0K-1}:\{\theta^{\left(K-1\right)}\leq 0\}$ and the global null hypothesis is defined as $H_{0}:\{\theta^{\left(1\right)}=\theta^{\left(2\right)}=\cdots =\theta^{\left(K-1\right)}=0\}$. Let $\eta_{j},\epsilon_{j}$ be the upper and lower critical values at stage j (with $\eta_{J}=\epsilon_{J}$) that are used for the decision‐making throughout the trial (as described below).

Consider the following Bayesian model:

$$\begin{array}{ll} & Y_{i}^{\left(k\right)}∼N(\mu^{\left(k\right)},\tau^{-1})\;k=0,\ldots ,K-1\;\text{and}\;i=1,\ldots ,n_{j}^{\left(k\right)}\\ & \mu^{\left(1\right)}∼N(\mu_{0}^{\left(1\right)},\tau_{01}^{-1}),\;\mu^{\left(0\right)}∼N(\mu_{0}^{\left(0\right)},\tau_{00}^{-1})\\ & \delta^{\left(k\right)}∼N(\delta_{0}^{\left(k\right)},{\left(\tau_{d}^{\left(k\right)}\right)}^{-1}),\;\mu^{\left(k+1\right)}=\mu^{\left(k\right)}-\delta^{\left(k\right)}\;k=1,\ldots ,K-2. \end{array}$$

If $\delta^{\left(k\right)},k\in \{1,\ldots K-2\}$ are, for instance, restricted to be positive random variables, then the treatment means are assumed to be ordered: $\mu^{\left(1\right)}\geq \ldots \geq \mu^{\left(K-1\right)}$. However, in this setting, we assume that there is uncertainty in the order of the treatment means effects and we consider a normal prior distribution for $\delta^{\left(k\right)}$. This parametrization allows to correlate the treatment means to each other and to capture the uncertainty of the treatment means effects order through the distribution of $\delta^{\left(k\right)}$. Alternative parametrization strategies are discussed in Section 5.

The joint distribution of the response means is normally distributed with mean $\mu =(\mu_{0}^{\left(1\right)},\ldots ,\mu_{0}^{\left(s\right)},\ldots ,\mu_{0}^{\left(K-1\right)})$, where $\mu_{0}^{\left(s\right)}=\mu_{0}^{\left(1\right)}-\sum_{r=1}^{s-1}\delta_{0}^{\left(r\right)},s\in \{2,\ldots ,K-1\}$ and covariance matrix Ω. The covariance matrix has diagonal elements defined as $\mathrm{Var}\left(\mu_{0}^{\left(s\right)}\right)=\tau_{01}^{-1}+\sum_{r=1}^{s-1}{\left(\tau_{d}^{\left(r\right)}\right)}^{-1},s\in \{2,\ldots ,K-1\}$ and the non‐diagonal element at row s and column t with s,t∈{1,…K−1}, is $\mathrm{Var}\left(\mu_{0}^{\left(\min(s,t)\right)}\right)$. The posterior distribution of the treatment means given the cumulative data up to stage j is

$$\left(\begin{array}{c} \mu^{\left(1\right)}\\ \cdot \\ \cdot \\ \mu^{\left(K-1\right)} \end{array}\right)|\bar{Y_{j}}∼N\left(\Gamma_{j}^{-1}a_{j},\Gamma_{j}^{-1}\right),$$

with $\Gamma_{j}=\Omega^{-1}+n_{j}\sum^{-1},\;a_{j}=\Omega^{-1}\mu +\sum^{-1}n_{j}{\bar{Y}}_{j}$.

At each interim analysis, the decision to stop the trial for efficacy or to drop the unpromising arms is based on the evaluation of the posterior probability of the treatment effect given the data up to stage j. At each stage, the posterior probability that the treatment effect relative to arm k is greater than zero, $P(\theta^{\left(k\right)}>0|\bar{Y_{j}})$, is computed. If this probability is below a pre‐specified threshold, $\epsilon_{j}$, that is, $P(\theta^{\left(k\right)}>0|\bar{Y_{j}})<\epsilon_{j}$ then treatment arm k is dropped. Arm k, instead, can be declared as superior to control at stage j if the posterior probability of the treatment effect being greater than zero given the observed data is larger than a specific threshold, $\eta_{j}$, that is, $P(\theta^{\left(k\right)}>0|\bar{Y_{j}})\geq \eta_{j}.$

If the posterior probability falls inside the two bounds, $\epsilon_{j}\leq P(\theta^{\left(k\right)}>0|\bar{Y_{j}})<\eta_{j}$, then the decision to proceed further with arm k and control is made as there is not enough information to decide whether to stop the arm or to claim its efficacy. Differently from the conventional frequentist MAMS design,^3^ the trial can still continue when an arm has crossed the upper bound and is stopped for efficacy. In this case, all other treatment arms remaining in the trial can proceed to the next stage. In this way, a decision on each treatment arm can be made at the end of the study, and the power to reject all hypotheses is increased.

### 3.1 Family‐wise error rate and power requirements

In confirmatory clinical trials, the control of the family‐wise error rate (FWER) is often required.^22^ For the proposed design, we ensure weak control of the FWER under the global null and it provides the control of the FWER under the partial null hypotheses (see details below). While the design might not guarantee control of the FWER under the entire null space, it can benefit from the Bayesian framework while still providing protection from erroneous conclusions^23^ under particular regions of the null space.

The critical bounds $\eta_{j},\epsilon_{j}$ can be chosen in order to maintain such control at level α. The FWER under the global null can be written as

(1) $$\begin{array}{ll} & P(\text{rejecting at least one true}\;H_{0k},\;k\in \{1,\ldots ,K-1\}|H_{0})\\ & \;\;=P(P(\theta^{\left(k\right)}>0|\bar{Y_{j}})\geq \eta_{j}\;\text{for some}\;(k,j)\in \{1,\ldots ,K-1\}\times \{1,\ldots ,J\}|H_{0}). \end{array}$$

Define $A_{k,j}=\{P(\theta^{\left(k\right)}>0|\bar{Y_{j}})<\epsilon_{j}\}$ and $B_{k,j}=\{\epsilon_{j}\leq P(\theta^{\left(k\right)}>0|\bar{Y_{j}})<\eta_{j}\}.$ If the event $A_{k,j}$ occurs then the treatment arm k is dropped for futility at stage j. If instead $B_{k,j}$ is satisfied, the arm k can proceed to stage j+1. The event that all hypotheses $H_{01},\ldots ,H_{0K-1}$ fail to be rejected can be defined as

$$R_{K-1}={⋂}_{k=1}^{K-1}\left({⋃}_{j=1}^{J}\left[\left\{{⋂}_{i=1}^{j-1}B_{k,i}\right\}\cup A_{k,j}\right]\right).$$

It follows that

(2) $$P(\text{rejecting at least one true}\;H_{0k},\;k\in \{1,\ldots ,K-1\}|H_{0})=1-P(R_{K-1}|H_{0}).$$

The objective of the study is to identify all promising treatment arms. Thus, the design is powered to reject all hypotheses for which the true treatment effects are different from zero: $\theta =(\theta^{\left(1\right)},\ldots ,\theta^{\left(K-1\right)})$, with $\theta^{\left(1\right)},\ldots ,\theta^{\left(K-1\right)}>0$. Using the definition

$${\bar{R}}_{K-1}={⋂}_{k=1}^{K-1}\left({⋃}_{j=1}^{J}\left[\left\{{⋂}_{i=1}^{j-1}B_{k,i}\right\}\cup \bar{\left\{A_{k,j}\cup B_{k,j}\right\}}\right]\right),$$

the power to reject all hypotheses is equal to $P({\bar{R}}_{K-1}|\theta )$.

Below, we provide the example on what form the quantities introduced above take under the setting of the motivating 3‐arm 2‐stage trial.

#### 3.1.1 3‐arm 2‐stage

For the 3‐arm 2‐stage trial, the event that all hypotheses fail to be rejected is

$$\begin{array}{ll} R_{2} & =\left\{\left\{\{P(\theta^{\left(1\right)}>0|\bar{Y_{1}})<\epsilon_{1}\}\cap \{P(\theta^{\left(2\right)}>0|\bar{Y_{1}})<\epsilon_{1}\}\right\}\right.\\ & \cup \{\{P(\theta^{\left(1\right)}>0|\bar{Y_{1}})<\epsilon_{1}\}\cap \{\epsilon_{1}\leq P(\theta^{\left(2\right)}>0|\bar{Y_{1}})<\eta_{1}\}\cap \{P(\theta^{\left(2\right)}>0|\bar{Y_{2}})<\epsilon_{2}\}\}\\ & \cup \{\{P(\theta^{\left(2\right)}>0|\bar{Y_{1}})<\epsilon_{1}\}\cap \{\epsilon_{1}\leq P(\theta^{\left(1\right)}>0|\bar{Y_{1}})<\eta_{1}\}\cap \{P(\theta^{\left(1\right)}>0|\bar{Y_{2}})<\epsilon_{2}\}\}\\ & \left.\cup \{\{\epsilon_{1}\leq P(\theta^{\left(2\right)}>0|\bar{Y_{1}})<\eta_{1}\}\cap \{P(\theta^{\left(2\right)}>0|\bar{Y_{2}})<\epsilon_{2}\}\cap \{\epsilon_{1}\leq P(\theta^{\left(1\right)}>0|\bar{Y_{1}})<\eta_{1}\}\cap \{P(\theta^{\left(1\right)}>0|\bar{Y_{2}})<\epsilon_{2}\}\}\right\}. \end{array}$$

Below, we describe how to search for the critical bounds $\eta_{j},\epsilon_{j},j\in \{1,\ldots ,J\}$ and the sample size in order to satisfy the FWER and the power requirements.

### 3.2 Search of the critical bounds and the sample size

To control the FWER at level α under the global null hypothesis and to power the design at 1−β under the configuration θ, the following inequalities need to be satisfied

(3) $$1-P(R_{K-1}|H_{0})\leq \alpha ,\;\text{and}\;P({\bar{R}}_{K-1}|\theta )\geq (1-\beta ).$$

Firstly, consider how to compute the single probability events $P(A_{k,j})$ and $P(B_{k,j})$. It can be seen that the inequalities $P(\theta^{\left(k\right)}>0|\bar{Y_{j}})<\epsilon_{j}$ and $\epsilon_{j}\leq P(\theta^{\left(k\right)}>0|\bar{Y_{j}})<\eta_{j}$ are satisfied if

$$\frac{𝔼\left[\mu_{|\bar{Y_{j}}}^{\left(k\right)}\right]-𝔼\left[\mu_{|{\bar{Y}}_{j}^{\left(0\right)}}^{\left(0\right)}\right]}{\sqrt{\mathrm{Var}\left(\mu_{|\bar{Y_{j}}}^{\left(k\right)}\right)+\mathrm{Var}\left(\mu_{|{\bar{Y}}_{j}^{\left(0\right)}}^{\left(0\right)}\right)}}<z_{\epsilon_{j}}\;\text{and}\;z_{\epsilon_{j}}\leq \frac{𝔼\left[\mu_{|\bar{Y_{j}}}^{\left(k\right)}\right]-𝔼\left[\mu_{|{\bar{Y}}_{j}^{\left(0\right)}}^{\left(0\right)}\right]}{\sqrt{\mathrm{Var}\left(\mu_{|\bar{Y_{j}}}^{\left(k\right)}\right)+\mathrm{Var}\left(\mu_{|{\bar{Y}}_{j}^{\left(0\right)}}^{\left(0\right)}\right)}}<z_{\eta_{j}},$$

respectively, where $z_{\eta_{j}},z_{\epsilon_{j}}$ are the $\eta_{j},\epsilon_{j}\text{‐}$percentiles of a standard normal. Given the Bayesian model described in Section 3 it follows that

$$𝔼\left[\mu_{|{\bar{Y}}_{j}^{\left(0\right)}}^{\left(0\right)}\right]=\frac{\mu_{0}^{\left(0\right)}\tau_{00}+{\bar{Y}}_{j}^{\left(0\right)}I_{j}^{\left(0\right)}}{\tau_{00}+I_{j}^{\left(0\right)}},\;I_{j}^{\left(0\right)}=\frac{n_{j}^{\left(0\right)}}{\sigma_{0}^{2}},\;\mathrm{Var}\left(\mu_{|{\bar{Y}}_{j}^{\left(0\right)}}^{\left(0\right)}\right)=\frac{1}{\tau_{00}+I_{j}^{\left(0\right)}},$$

$$𝔼\left[\mu_{|\bar{Y_{j}}}^{\left(k\right)}\right]=\sum_{i=1}^{K-1}c_{ij}^{\left(k\right)}{\bar{Y}}_{j}^{\left(i\right)}+\gamma_{j}^{\left(k\right)},\;c_{ij}^{\left(k\right)}=\left(\Gamma_{j}^{-1}\sum^{-1}n_{j}\right)[k,i],\;\gamma_{j}^{\left(k\right)}=\left(\Gamma_{j}^{-1}\Omega^{-1}\mu \right)[k],\;\;\mathrm{Var}\left(\mu_{|\bar{Y_{j}}}^{\left(k\right)}\right)=(\Gamma_{j}^{-1})[k,k],$$

where the notation [k,i] indicates the element of the matrix at row k and column i, while [k] refers to the *k*th position in the vector. Thus, the inequality $P(\theta^{\left(k\right)}>0|\bar{Y_{j}})<\epsilon_{j}$ translates into solving

$$\overset{K-1}{\underset{i=1}{\sum }}c_{ij}^{\left(k\right)}{\bar{Y}}_{j}^{\left(i\right)}-\frac{{\bar{Y}}_{j}^{\left(0\right)}I_{j}^{\left(0\right)}}{\tau_{00}+I_{j}^{\left(0\right)}}<-\gamma_{j}^{\left(k\right)}+\frac{\mu_{0}^{\left(0\right)}\tau_{00}}{\tau_{00}+I_{j}^{\left(0\right)}}+z_{\epsilon_{j}}\sqrt{\mathrm{Var}\left(\mu_{|\bar{Y_{j}}}^{\left(k\right)}\right)+\mathrm{Var}\left(\mu_{|{\bar{Y}}_{j}^{\left(0\right)}}^{\left(0\right)}\right)},$$

and the distribution of the linear combination of the outcome variable means is

(4) $$\overset{K-1}{\underset{i=1}{\sum }}c_{ij}^{\left(k\right)}{\bar{Y}}_{j}^{\left(i\right)}-c_{j}^{\left(0\right)}{\bar{Y}}_{j}^{\left(0\right)}∼N\left(\overset{K-1}{\underset{i=1}{\sum }}c_{ij}^{\left(k\right)}\mu^{\left(i\right)}-c_{j}^{\left(0\right)}\mu^{\left(0\right)},\overset{K-1}{\underset{i=1}{\sum }}\frac{{\left(c_{ij}^{\left(k\right)}\sigma_{i}\right)}^{2}}{n_{j}^{\left(i\right)}}+\frac{{\left(c_{j}^{\left(0\right)}\sigma_{0}\right)}^{2}}{n_{j}^{\left(0\right)}}\right),$$

where $c_{j}^{\left(0\right)}=\frac{I_{j}^{\left(0\right)}}{\tau_{00}+I_{j}^{\left(0\right)}}$.

Thus, the probability of the event $A_{k,j}$ becomes $P(A_{k,j})=F\left(-\gamma_{j}^{\left(k\right)}+\frac{\mu_{0}^{\left(0\right)}\tau_{00}}{\tau_{00}+I_{j}^{\left(0\right)}}+z_{\epsilon_{j}}v_{j}^{k}\right),$ where $v_{j}^{k}=\sqrt{\mathrm{Var}\left(\mu_{|\bar{Y_{j}}}^{\left(k\right)}\right)+\mathrm{Var}\left(\mu_{|{\bar{Y}}_{j}^{\left(0\right)}}^{\left(0\right)}\right)}$ and F(x) is the cumulative density function of the normal distribution in (4). Similarly, the probability of the event $B_{k,j}$ can be computed.

When multiple arms are concurrently studied, the joint distribution of the probability events is a multivariate normal distribution and the covariance matrix changes depending on the number of arms that have reached a particular stage. For instance, the covariance between arms s,t at stage j and $j^{⋆},\;j\leq j^{⋆}$ is

$$\text{Cov}\left(\overset{K-1}{\underset{i=1}{\sum }}c_{ij}^{\left(s\right)}{\bar{Y}}_{j}^{\left(i\right)}-c_{j}^{\left(0\right)}{\bar{Y}}_{j}^{\left(0\right)},\overset{K-1}{\underset{i=1}{\sum }}c_{ij^{⋆}}^{\left(t\right)}{\bar{Y}}_{j^{⋆}}^{\left(i\right)}-c_{j^{⋆}}^{\left(0\right)}{\bar{Y}}_{j^{⋆}}^{\left(0\right)}\right)=\overset{K-1}{\underset{i=1}{\sum }}c_{ij}^{\left(s\right)}c_{ij^{⋆}}^{\left(t\right)}\text{Cov}({\bar{Y}}_{j}^{\left(i\right)},{\bar{Y}}_{j^{⋆}}^{\left(i\right)})+(c_{j}^{\left(0\right)}c_{j^{⋆}}^{\left(0\right)})\mathrm{Var}({\bar{Y}}_{j^{⋆}}^{\left(0\right)}),$$

where $\text{Cov}({\bar{Y}}_{j}^{\left(i\right)},{\bar{Y}}_{j^{⋆}}^{\left(i\right)})=t_{j}\mathrm{Var}({\bar{Y}}_{j}^{\left(i\right)})$ if treatment i has reached stage $j^{⋆}\neq j$ and the interim analysis is done at $t_{j}\%$ of the total population, otherwise $\text{Cov}({\bar{Y}}_{j}^{\left(i\right)},{\bar{Y}}_{j^{⋆}}^{\left(i\right)})=\mathrm{Var}({\bar{Y}}_{j}^{\left(i\right)})$, with $\mathrm{Var}({\bar{Y}}_{j}^{\left(i\right)})=\frac{\sigma_{i}^{2}}{n_{j}^{\left(i\right)}}$.

In order to compute $P(R_{K-1})$, for each event in $R_{K-1}$, one needs to find $c_{ij}^{\left(k\right)},\gamma^{\left(k\right)}$ and the covariance matrix of the multivariate normal distribution. The critical values can be expressed as a function of a parameter, p, $\epsilon_{j}=\epsilon_{j}(p),\eta_{j}=\eta_{j}(p)$, so that the search is restricted to a grid of values for p with $\epsilon_{j}(p),\eta_{j}(p)\in [0,1]$ and the sample size $n_{j}$ in order to satisfy the inequalities in (3). Additional constraints can be considered in order to maintain control of the FWER under the partial null hypotheses. In the 3‐arm setting, we use these partial null configurations: $\left(\theta^{\left(1\right)},0\right)$ and $\left(0,\theta^{\left(2\right)}\right)$. However, it can be observed that the FWER computed under the global null hypothesis depends on the value of $\mu^{\left(0\right)}$. As outlined in Stallard et al,^24^ control of the type I error is not possible for every value of $\mu^{\left(0\right)}$. Indeed, under the hypothesis that $\mu^{\left(i\right)}=\mu^{\left(0\right)},i\in \{1,\ldots ,K-1\}$, if for example $\mu^{\left(0\right)}\to \infty$ and $\sum_{i=1}^{K-1}c_{ij}^{\left(k\right)}-c_{j}^{\left(0\right)}>0$ then the expected value of the normal distribution in Equation (4) goes to infinity and thus in this case it is not possible to control the FWER at level α. Thus, the critical bounds can be found for specific values of the treatment means under which the FWER is controlled. Exploratory analyses can then be conducted in order to explore the operating characteristics of the design for different true values of the control mean.

The description of the probability events to compute the FWER for the special case of a 3‐arm 2‐stage design is described in detail in Section 1 of Data S1.

In the next section, an extensive simulation study will be performed in order to evaluate the operating characteristics of the proposed design in the setting of the motivating trial.^20^

## 4 CASE STUDY REVISITED

### 4.1 Setting

We revisit the results of the clinical trial of *Tiotropium add‐on therapy in adolescents with moderate asthma: A 1‐year randomized controlled trial* [NCT01257230]^20^ using prior information from the study *Tiotropium in asthmatic adolescents symptomatic despite inhaled corticosteroids: A randomised dose‐ranging study* [NCT01122680]^21^ introduced in Section 2.

In the phase III study (NCT01257230), patients were randomized in a 1:1:1 ratio to receive 5μg or 2.5μg of once‐daily tiotropium or placebo. The null hypotheses were tested in a stepwise manner to control the type I error at level α=0.025. The study was powered at 80% to detect a difference of 120 mL between treatments in the change from baseline of peak FEV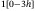 assuming a common standard deviation of 340 mL. It was found that 127 patients per group were needed, resulting in a maximum sample size of 381 patients. The trial is revisited using the proposed Bayesian design and the prior distributions are constructed using information from the previous study (NCT01122680).

In line with the Phase III trial, we assume that the change from baseline of peak FEV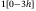 is normally distributed with standard deviation $\sigma_{k}=340,k\in \{0,1,2\}$. For the prior distributions, we set $\mu_{0}^{\left(0\right)}=489$ and $\mu_{0}^{\left(1\right)}=602$, as these are the estimated mean responses obtained at the end of the Phase II NCT01122680 study. We center the prior distribution of $\delta^{\left(1\right)}$ at 0, so that a‐priori we do not assume a difference between the treatment means. Given the lack of information regarding the between‐patient variability, we firstly consider uninformative priors with $\tau_{00}=\tau_{01}=\tau_{d}^{\left(1\right)}=10^{-6}$. Additional analyses are then conducted in order to explore how the design performs with more informative prior distributions.

For the simulation scenarios, we set the true $\mu^{\left(0\right)}$ to be equal to the estimated mean response obtained at the end of Phase II, that is $\mu^{\left(0\right)}=489$, while for the treatment arms $\mu^{\left(k\right)}=\mu^{\left(0\right)}+\theta^{\left(k\right)},k\in \{1,2\}$. As in the original study, we consider an improvement of ${\text{FEV}}_{1}$ of 120 of interest. The trial is then designed to achieve 80% power under the configuration θ=(120,120).

We evaluate the performance of the design under various treatment effect configurations when a monotonic order is assumed to be true: $\theta^{\left(1\right)}=120\;\text{and}\;\theta^{\left(2\right)}\in \{0,20,40,60,80,120\}$ and the configurations where the order is violated: $\theta^{\left(2\right)}=120\;\text{and}\;\theta^{\left(1\right)}\in \{0,20,40,60,80,120\}$.

The sample size and the critical bounds are found in order to reject all hypotheses at 80% when θ=(120,120) with $\mu^{\left(1\right)}=609,\mu^{\left(2\right)}=609,\mu^{\left(0\right)}=489$ and to control the FWER at level α=0.025 under $H_{0}=\{\mu^{\left(1\right)}=\mu^{\left(2\right)}=\mu^{\left(0\right)}=489\}$ and two partial nulls $H_{01}=\{\mu^{\left(1\right)}=\mu^{\left(0\right)}=489,\mu^{\left(2\right)}=609\}$ and $H_{02}=\{\mu^{\left(1\right)}=609,\mu^{\left(2\right)}=\mu^{\left(0\right)}=489\}$. A single interim analysis after half of the total study population is observed and triangular^25^ critical bounds are used, that is $\eta_{2}=\epsilon_{2}=\Phi (\frac{\Phi^{-1}(\eta_{1})}{\left(1+0.5\right)}\frac{2}{\sqrt{2}}),\epsilon_{1}=\Phi (\frac{\Phi^{-1}(\eta_{1})}{\left(1+0.5\right)}\frac{1}{2})$, where Φ(·) is the cumulative density function of a standard normal distribution. Additionally to the achieved power, the efficiency of the proposed design is measured by its expected sample size (ESS), that is the mean number of patients recruited to the trial before it is terminated.

The proposed Bayesian design is compared to the frequentist ORD,^5^ developed under an order assumption among the treatment effects, the modified MAMS design, MAMS(m),^3^ whose modification allows all treatment arms that have not crossed any bounds to continue to the next stage even when one arm crossed the upper boundary, and the frequentist design proposed by Urach and Posch^26^ (Urach & Posch) with separate stopping rules and futility critical boundaries which apply the closure principle to define a sequentially rejective group sequential test.

All designs use triangular critical bounds^25^ and the numerical results are found using R^27^ and $10^{4}$ replicate simulations. In the simulations, the posterior distributions are estimated using MCMC samples through the package rjags.^28^ The critical bounds and the sample size for the Bayesian design are found computing all probabilities to reject at least one null hypothesis under the global null and the probability to reject both hypotheses under the partial null configurations as described in Sections 3.1 and 3.2, without the use of MCMC sampling for the posterior distributions. The package mvtnorm ^29^ is used to compute the integrals in R.^27^ Results using Pocock^30^ critical bounds are provided in Section 2 of Data S1.

### 4.2 Numerical results

We start by considering the Bayesian design with no prior information for the treatment means and the treatment mean difference, that is $\tau_{00}=\tau_{01}=\tau_{d}^{\left(1\right)}=10^{-6}$. We refer to this version of the design as to B‐MAMS(U). The obtained design parameters and results of the simulations under the global null hypothesis for all considered approaches are given in Table 1. One can note that all considered designs control the FWER at level α=0.025 as required.

**TABLE 1:** Probability to reject at least one hypothesis (FWER), maximum sample size (Max SS) and expected sample size (ESS) for each design under the global null hypothesis $H_{0}$ with $\mu^{\left(1\right)}=\mu^{\left(2\right)}=\mu^{\left(0\right)}=489$ when all designs are powered at 80% to reject all hypotheses under θ=(120,120).

Design	$u_{1},u_{2},l_{1},\nu_{1},\nu_{2}$	$\eta_{1},\eta_{2},\epsilon_{1}$	Max SS	ESS	FWER
MAMS(m)	2.482, 2.34, 0.827, ‐, ‐		612	380.85	0.025
B‐MAMS(U)		0.9934, 0.9906, 0.7959	612	378.43	0.025
ORD	2.223, 2.095, 0.741, ‐, ‐		534	316.91	0.024
B‐MAMS(D)		0.9927, 0.9893, 0.7921	576	355.19	0.024
Urach & Posch	2.482, 2.34, 0.827, 2.222, 2.095		552	343.12	0.025
B‐MAMS(C)		0.9906, 0.9866, 0.7832	492	303.75	0.024

*Notes*: Results are provided using $10^{4}$ simulations. Upper—$u_{j},j\in \{1,2\}$—and lower—$l_{1}$—triangular critical bounds are provided for the ORD and MAMS(m) frequentist designs. For the Urach & Posch design $u_{j},j\in \{1,2\}$ and $l_{1}$ are the upper and lower global boundaries, while $\nu_{j},j\in \{1,2\}$ are the elementary boundaries.

The B‐MAMS(U) design requires a total maximum sample size of 612 patients, which is the same maximum total sample size required by the MAMS(m) to achieve 80% of power to reject both hypotheses. At the same time, the ORD and the Urach & Posch designs require a total of 534 and 552 patients, respectively.

One of the advantages of using a Bayesian design is that it allows incorporating historical information. One possibility is, for example, to include information on the treatment mean difference between the active treatment arms. Specifically, we use $\tau_{d}^{\left(1\right)}=5.9\times 10^{-5}$ (and $\tau_{00}=\tau_{01}=10^{-6}$ as before) and refer to this design option of the Bayesian design as to B‐MAMS(D). The Bayesian design B‐MAMS(D) can control the FWER at level α under the global and partial null hypotheses and the power to reject both hypotheses is 80%. This design requires a total maximum sample size of 576 patients, which is higher compared to the ORD and Urach & Posch designs—for these 42 and 24 fewer patients are required respectively—but lower compared to the MAMS(m) design.

Another possibility is to include prior information on the control arm. We refer to this design as to B‐MAMS(C) and we consider a prior on control that is worth an extra 20% of data compared to the frequentist MAMS(m) design. If $\tau_{00}=0.00039,\tau_{01}=\tau_{d}^{\left(1\right)}=10^{-6}$, then the Bayesian design would require 82 patients per arm per stage that leads to a total maximum sample size of 492 patients compared to 612 patients in the frequentist MAMS(m) design and 534 for the ORD design.

**FIGURE 1:**
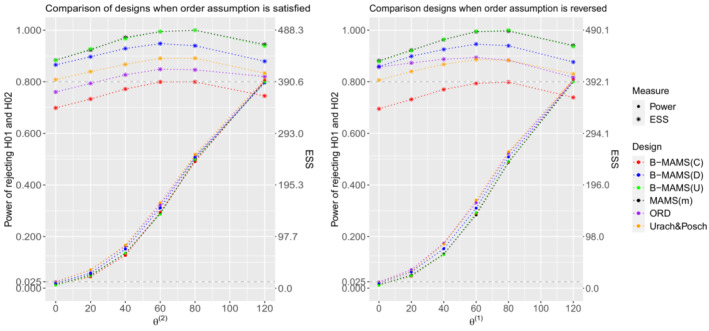
Power to reject all hypotheses and expected sample sizes (ESS) under $\theta =(120,\theta^{\left(2\right)})$ and $\theta^{\left(2\right)}\in \{0,20,40,60,80,120\}$ (left) and under $\theta =(\theta^{\left(1\right)},120)$ and $\theta^{\left(1\right)}\in \{0,20,40,60,80,120\}$ (right) for the 3‐arm 2‐stage MAMS(m), ORD, Urach & Posch and Bayesian designs when all designs are powered at 80% to reject both hypotheses under θ=(120,120). All designs use triangular bounds. Results are provided using $10^{4}$ replications.

**FIGURE 2:**
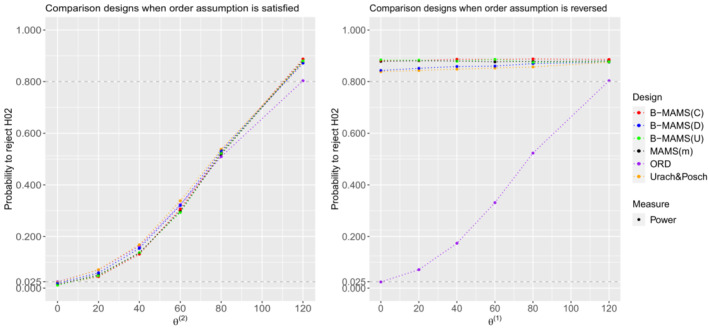
Probability to reject $H_{02}$ under $\theta =(120,\theta^{\left(2\right)})$ and $\theta^{\left(2\right)}\in \{0,20,40,60,80,120\}$ (left) and under $\theta =(\theta^{\left(1\right)},120)$ and $\theta^{\left(1\right)}\in \{0,20,40,60,80,120\}$ (right) for the 3‐arm 2‐stage MAMS(m), ORD, Urach & Posch and Bayesian designs when all designs are powered at 80% to reject both hypotheses under θ=(120,120). All designs use triangular bounds. Results are provided using $10^{4}$ replications.

The designs' performances under the non‐global null configurations are given in Figures 1 and 2 showing the probability to reject both hypotheses and the second hypothesis, respectively. Note that, when the first or the second treatment are no different from control, $\theta^{\left(1\right)}=0$ or $\theta^{\left(2\right)}=0$, the probability to reject all hypotheses is controlled at level α for all considered designs.

It can be observed that the B‐MAMS(U) design matches the operating characteristics of the frequentist MAMS(m) design. Indeed, for all considered scenarios the two designs have the same probability to reject both hypotheses and the same probability to reject the second hypothesis $H_{02}$. The two designs provide also the same ESS under all considered scenarios. Compared to the other two frequentist approaches, when the order is satisfied, the B‐MAMS(U) design shows lower power of rejecting both hypotheses—a difference up to 4.4% and 3.6% compared to the Urach & Posch and ORD designs respectively (see the left panel in Figure 1). As shown on the left panel in Figure 2, the probability to reject the second hypothesis is smaller for the B‐MAMS(U) design when $\theta^{\left(2\right)}\leq 80$ compared to the Urach & Posch design—a difference up to 4.4%—and smaller compared to the ORD when $\theta^{\left(2\right)}\leq 60$—a difference up to around 3%. When $\theta^{\left(2\right)}=120$, the B‐MAMS(U) shows an increase of around 7% in the probability to reject the second hypothesis compared to the ORD. In terms of ESS, the B‐MAMS(U) shows an increase up to 12% and 18% depending on the scenario compared to the Urach & Posch and ORD designs, respectively, when the assumption on the treatment effect order is satisfied. When this assumption is violated, the B‐MAMS(U) has lower power—a difference up to 4.9% and 4.3% compared to the Urach & Posch and ORD designs—to reject both hypotheses. However, the probability to reject the second hypothesis is higher for the B‐MAMS(U) design compared to the Urach & Posch and ORD designs—an increase up to 4.5% and 86%, respectively depending on the simulation scenario (right panel in Figure 2). In terms of ESS, the B‐MAMS(U) shows an increase up to 13% and 14% depending on the scenario compared to the Urach & Posch and ORD designs, respectively.

When the order is satisfied, the probability to reject both hypotheses for the B‐MAMS(D) is slightly lower—a difference up to around 1.2% and 2%—compared to the frequentist ORD and Urach & Posch designs respectively, while it is slightly higher compared to the MAMS(m)—an increase up to 2%. The B‐MAMS(D) design shows higher probability to reject the second hypothesis compared to the ORD when $\theta^{\left(2\right)}\geq 80$—an increase of around 3% and 7% when $\theta^{\left(2\right)}=80$ and $\theta^{\left(2\right)}=120$, respectively. However, the probability to reject the second hypothesis is slightly lower compared to the Urach & Posch design (see plot on the left in Figure 2). In addition, the B‐MAMS(D) design has lower ESS compared to the MAMS(m)—a reduction up to 6.8% depending on the simulation scenario, while it has higher ESS compared to the ORD and Urach & Posch designs—up to 13% and 7%, respectively, depending on the scenario when the order assumption is satisfied. When the order is reversed, the probability to reject the second hypothesis is higher for the B‐MAMS(D) design: it is above 80% and slightly higher compared to the Urach & Posch design, while it is noticeably smaller for the ORD. However, the B‐MAMS(D) design shows a decrease up to around 2% and 3% compared to the frequentist ORD and Urach & Posch designs respectively in the probability to reject both hypotheses. The increase in power for the frequentist designs is due to the hierarchical testing procedure that is not used in the proposed Bayesian designs.

For all the considered scenarios, the probability to reject both hypotheses is almost the same for the B‐MAMS(C) and the MAMS(m) designs independently of the order assumption. However, the total maximum sample size for the Bayesian design is 20% smaller compared to the frequentist design. The B‐MAMS(C) provides advantages to reject the second hypothesis compared to all other designs when the order assumption is reversed. Indeed, the B‐MAMS(C) has the highest probability to reject the second hypothesis and the smallest ESS compared to all other designs for all considered scenarios—a reduction in ESS up to 14%, 19%, and 20% compared to the Urach & Posch, ORD and MAMS(m) designs respectively.

Overall, the results suggest that the proposed Bayesian design provides benefits compared to the considered frequentist approaches in terms of ESS—the B‐MAMS(C) provides a reduction up to 14%, 19%, and 20% compared to the Urach & Posch, ORD, and MAMS(m) designs, respectively, while the B‐MAMS(D) provides a reduction up to 6.8% compared to the MAMS(m) design—and probability to reject the second hypothesis—an increase up to around 1.3%, 4.2%, and 4.6% for the B‐MAMS(D), B‐MAMS(C), and B‐MAMS(U) compared to the Urach & Posch and an increase up to 86% compared to the ORD—when the order assumption is not satisfied. Nevertheless, the proposed design shows a reduction in power to reject both hypotheses—the B‐MAMS(D) shows a difference up to around 2% and 3% compared to the ORD and Urach & Posch designs, while the B‐MAMS(C) and B‐MAMS(U) provide both a difference of around 5% and 4.3% compared to the Urach & Posch and ORD designs respectively. In addition, when historical information is disregarded, the proposed design can match the operating characteristics of the MAMS(m) frequentist design.

### 4.3 Explorations of the FWER control

In the simulation studies described above, $\mu^{\left(0\right)}$ was assumed to be equal to the true value mean on control. In this section, we investigate the robustness of the results when the true value and the prior mean on control differ.

**FIGURE 3:**
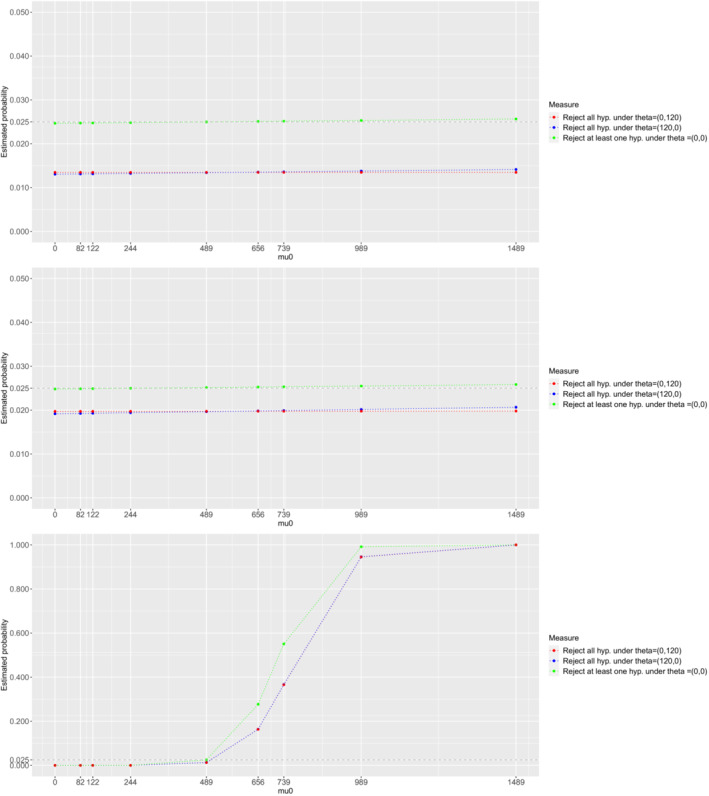
Probability to reject at least one $H_{0k}$ under θ=(0,0) and probability to reject all hypotheses under θ=(120,0) and θ=(0,120) for the 3‐arm 2‐stage Bayesian design when it is powered at 80% to reject both hypotheses under θ=(120,120). Figure at the top refers to the B‐MAMS(U) design which incorporates no prior information on the treatment means—prior of $\mu^{\left(0\right)}$ is centered in $\mu_{0}^{\left(0\right)}=489$ and has standard deviation $\sigma =\sqrt{\frac{1}{\tau_{00}}}=1000$. Figure in the middle refers to the B‐MAMS(D) design. Figure at the bottom refers to the B‐MAMS(C) design which incorporates prior information on the control mean—prior of $\mu^{\left(0\right)}$ is centered in $\mu_{0}^{\left(0\right)}=489$ and has standard deviation $\sigma =\sqrt{\frac{1}{\tau_{00}}}=50.63$. 3‐arm 2‐stage designs use triangular bounds.

In Figure 3, we explore the probabilities to reject at least one null hypothesis under the global null (ie, θ=(0,0)) and the probability to reject both hypotheses under the partial null configurations (ie, θ=(120,0) and θ=(0,120)) for several values of the mean of the control arm ($\mu^{\left(0\right)}$). The numerical results are provided using an analytical expression—computing all probabilities as described in Sections 3.1 and 3.2, without the use of MCMC sampling for the posterior distributions.

It can be observed that, for the B‐MAMS(U) and the B‐MAMS(D), the top and the middle panel of Figure 3, respectively, the FWER under the global null hypothesis is at most around 2.6% (an inflation of around 0.1%) when the true mean is off the prior mean by a factor of 3, $\mu^{\left(0\right)}=1489$, whereas the probability to reject both hypotheses under θ=(0,120) and θ=(120,0) is controlled for both designs—it is below 2% for all values of $\mu^{\left(0\right)}$. The bottom panel of Figure 3 shows how the probability to reject at least one or all hypotheses changes when $\mu^{\left(0\right)}$ is shifted from the true prior mean for the B‐MAMS(C) design. It can be observed that the FWER under the global null hypothesis is sharply increasing for the considered values of $\mu^{\left(0\right)}$, reaching around 27% when the true mean is off the prior mean by a factor of 1.3, $\mu^{\left(0\right)}=656$, whereas the probability to reject both hypotheses under the partial null configurations is around 16% for $\mu^{\left(0\right)}=656$. This design shows major inflations on the FWER because it is the one that considers an informative prior distribution on the control mean and thus requires the least sample size compared to all other designs.

**FIGURE 4:**
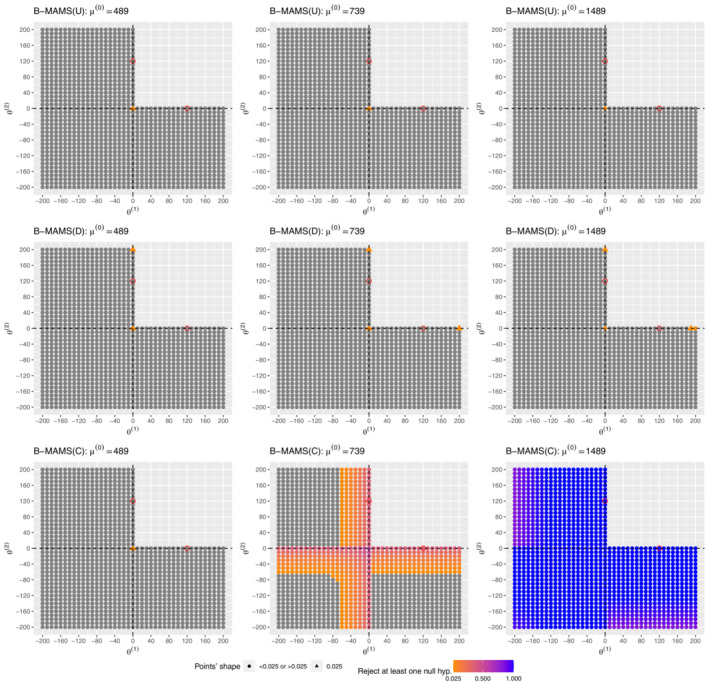
Probability to reject at least one null hypothesis when $\mu^{\left(0\right)}=489,739,1489$ for different values of the treatment effects for the 3‐arm 2‐stage B‐MAMS(C), B‐MAMS(D), B‐MAMS(U) designs when they are powered at 80% to reject both hypotheses under θ=(120,120). Points in grey colour refer to probability values below 0.025. Partial null configurations—θ=(120,0) and θ=(0,120)—are marked with red circles.

Figure 4 provides further explorations of the probability to reject at least one null hypothesis for three values of the control mean, $\mu^{\left(0\right)}=489,739,1489$, but for varying values of the treatment effects for the 3‐arm 2‐stage B‐MAMS(C), B‐MAMS(D), B‐MAMS(U) designs when they are powered at 80% to reject both hypotheses under θ=(120,120). Specifically, we investigate how the FWER behaves for several values of the treatment effects, $\theta^{\left(1\right)}$ and $\theta^{\left(2\right)}\in (-200,200)$.

It can be observed that the B‐MAMS(U) and B‐MAMS(D) designs (top and middle panels of Figure 4) are both robust in controlling the FWER around the global and partial null hypotheses for all the considered values of the control mean. Under the global null hypothesis the FWER is equal to 0.025 when the control mean is $\mu^{\left(0\right)}=489$ or 739, while it is slightly inflated—around 2.6%—when $\mu^{\left(0\right)}=1489$. The B‐MAMS(D) design shows also some minor inflations—around 0.1%—when the treatment effects are close to 200.

The B‐MAMS(C) shows major inflations for all considered values of the treatment effects around the global and partial nulls when the control mean is off by a factor between half and one standard deviation to the prior mean. These inflations are more pronounced for all considered treatment effects when the control mean is off by a factor of 3.

Overall, these exploratory analyses have shown minor inflations on the FWER when the true control mean is different from the prior assumption and the precision on the control mean is small. Thus, the proposed design can provide robust results when the assumption on the value of the control mean is violated and it provides benefits in terms of power compared to other frequentist approaches. However, there can be substantial inflation of the FWER when using an informative prior distribution on the control mean.

Nevertheless, the analyses presented were restricted to the setting of the case study. To evaluate the operating characteristics of the proposed design under different scenarios and possible misspecification of the order assumption compared to the motivating trial, we have conducted further analyses considering two more settings: one with more treatment arms, a 4‐arm 2‐stage trial, and one with more stages, a 3‐arm 3‐stage trial. For these settings, we have explored how the FWER behaves under different values of the control mean.

For the 4‐arm 2‐stage trial, we have considered the same clinical setting as for the case study, but with one more additional arm. We investigate how the FWER behaves for several values of the treatment effects, with $\theta^{\left(1\right)}$ and $\theta^{\left(2\right)}$ varying from −200 to 200 by steps of 40, while $\theta^{\left(3\right)}$ is varying from −200 to 200 by steps of 80. In this way, all possible misspecifications of the order effects can be explored. As for the 3‐arm 2‐stage setting, minor inflations (up to 0.7%) of the FWER are shown when the true control mean is different from the prior assumption and the precision on the control mean is small for the B‐MAMS(U) and B‐MAMS(D) designs. However, there can be substantial inflation of the FWER when using an informative prior distribution on the control mean for the B‐MAMS(C) design. For example, for this chosen design, it can be observed (Figure 8 in Section 3 of Data S1) that the probability to reject at least one hypothesis is almost 1 when $\theta^{\left(1\right)}$ or $\theta^{\left(2\right)}$ are equal to zero and $\theta^{\left(3\right)}=-200$. In addition, for the 4‐arm 2‐stage design, we have also included some results to compare how the design performs compared to the frequentist ordered and multi‐arm multi‐stage designs. The corresponding results are presented in Section 3 of Data S1.

For the 3‐arm 3‐stage design, we considered other scenarios and normalized treatment effects. For this design we have explored the probability to reject at least one null hypothesis when $\mu^{\left(0\right)}=0,0.5,1$ and with the following parameters for the Bayesian model: $\tau =1,\tau_{01}=10^{-6},\mu_{0}^{\left(0\right)}=0,\mu_{0}^{\left(1\right)}=0,\delta_{0}^{\left(1\right)}=0$. In Table 3 in Data S1 we provide the sample sizes and the critical bounds that are found to control the FWER under the global and partial nulls at level α=0.025 when $\mu^{\left(0\right)}=0$ and the designs are powered at 80% to reject all hypotheses under θ=(0.5,0.5). However, similar conclusions to the 3‐arm 2‐stage and 4‐arm 2‐stage designs can be drawn for all the considered variants of the proposed design. The complete results are provided in Section 4 of Data S1.

## 5 DISCUSSION

The aim of this study was to develop a MAMS design that can account for historical information and incorporate an order relationship among the treatment arms. In the proposed approach, we model the correlation among the treatment means through the use of prior distributions for the treatment mean difference among two consecutive treatment arms. Through simulations and theoretical results we show that the proposed approach can provide benefits compared to the frequentist ORD^5^ when the order assumption is not satisfied. For specific values of the model parameters, the Bayesian design can also match the operating characteristics of the frequentist approaches.

In the considered setting, we are interested in selecting all arms (eg, doses) that are efficacious compared to the standard one. We allow termination of one arm first (eg, for efficacy) and then continuation of the trial with the remaining arms. Indeed, in some multiple dose settings, it is not guaranteed that higher doses lead to increased efficacy compared to lower doses—see for example, the TAILoR study.^31^ Likewise, in the setting where multiple durations of the same treatment arm are compared against a standard regimen, one could assume that longer treatment durations lead to higher efficacy compared to the shortest ones. But there could be uncertainty about this assumption as the increase in side effects or compliance with the treatment can lead to reduced effects on the treatment efficacy. So, in this case, stopping shorter durations at interim and continuing with longer ones could be a reasonable choice. Conversely, if a monotonic order assumption among the treatment effects is certain—that is, higher doses (or longer treatment durations) lead to higher efficacy compared to lower doses (or shorter durations), then the order restricted multi‐arm multi‐stage design^5^ can be implemented.

The proposed design ensures weak control of the FWER under specific values of the control mean. While strong control of the FWER is desirable and recommended by the Food and Drug Administration,^32^ this is not a must—see for example, recent COVID‐19 trials such as the Coverage study.^33^ Moreover, strong control in a Bayesian framework is challenging in general as any prior distribution that give notable weight outside of the null space leads to inflation of the FWER unless very conservative thresholds under the global null are used. We believe that ensuring weak control is still important. It is however essential to understand how the FWER behaves under reasonable misspecifications of the design's assumptions through exploratory analyses. The proposed design has been shown to be robust in controlling the FWER for several plausible values of the control mean.

Alternative approaches can be considered for modelling the ordering among the treatment arms. For example, one could consider prior distributions on the difference between each treatment mean and the largest treatment mean, that is, $\mu^{\left(1\right)}$. This alternative parametrization could be easily implemented and linked to the one proposed in this paper if normal distributions are used for modelling the treatment mean differences. In addition, one could consider prior distributions on the treatment effects and their differences. However, also in this case one needs to incorporate some prior distribution on the control mean. The Bayesian model can also be adapted when a strict order can be assumed among the treatment means. One could, for instance, define the prior distributions of $\delta^{\left(k\right)},k\in \{1,\ldots ,K-2\}$ to be lognormal distributions. In this case, the treatment means will result ordered as $\mu^{\left(1\right)}\geq \ldots \geq \mu^{\left(K-1\right)}$.

In this study, we assumed that the variance of patients' responses is known. However, in practice this might not be true. Further work needs to be done in order to incorporate prior information about $\sigma_{k},k\in \{0,\ldots ,K-1\}$ into the model. In addition, in this work, we have just considered normally distributed outcomes. Further work is needed in order to extend this framework to non‐normally distributed endpoints.

## AUTHOR CONTRIBUTIONS

All authors have directly participated in the planning and execution of the presented work.

## CONFLICT OF INTEREST

The authors declare no potential conflict of interests.

## Supporting information




**Data S1.** Supporting information

## Data Availability

No datasets were generated or analyzed during the current study. Programming code for reproducing the numerical results is available at GitHub: https://github.com/OrderedRestrictedDesign/3arm2stageDesigns.
