# Supplementary material for: A Bayesian multi‐arm multi‐stage clinical trial design incorporating information about treatment ordering
Source: Stat Med. 2023 May 9;42(16):2841–54. doi: 10.1002/sim.9752 (PMC10962588; doi:10.1002/sim.9752)
Supplement: Supplementary file 1 — Data S1. Supporting information [file SIM-42-2841-s001.pdf]

Received: Added at production | Revised: Added at production | Accepted: Added at production

DOI: xxx/xxxx

ARTICLE TYPE

Supporting Information for 'A Bayesian multi-arm multi-stage clinical trial design incorporating information about treatment ordering'

Alessandra Serra*[1] | Pavel Mozgunov[1] | Thomas Jaki[1,2]

[1]MRC Biostatistics Unit, University of Cambridge, United Kingdom
[2]Faculty for Informatics and Data Science, University of Regensburg, Germany

Correspondence
*Corresponding author name, Email: alessandra.serra@mrc-bsu.cam.ac.uk

Present Address
This is sample for present address text this is sample for present address text

Summary

Multi-Arm Multi-Stage (MAMS) designs can notably improve efficiency in later stages of drug development, but they can be suboptimal when an order in the effects of the arms can be assumed. In this work, we propose a Bayesian multi-arm multi-stage trial design that selects all promising treatments with high probability and can efficiently incorporate information about the order in the treatment effects as well as incorporate prior knowledge on the treatments. A distinguishing feature of the proposed design is that it allows taking into account the uncertainty of the treatment effect order assumption and does not assume any parametric arm-response model. The design can provide control of the family-wise error rate under specific values of the control mean and we illustrate its operating characteristics in a study of symptomatic asthma. Via simulations, we compare the novel Bayesian design with frequentist multi-arm multi-stage designs and a frequentist ordered restricted design that does not account for the order uncertainty and demonstrate the gains in the sample sizes the proposed design can provide. We also find that the proposed design is robust to violations of the assumptions on the order.

KEYWORDS:
Adaptive Designs; Bayesian Inference; Infectious Diseases; Multi-Arm Multi-Stage; Order Restriction.

1 | FWER FOR A 3-ARM 2-STAGE DESIGN

Let consider a special case of the Bayesian design where two active treatment arms are compared to the control and one interim analysis is planned at half of the total population. In this setting,

$$
\begin{aligned}
R_2 = \Big\{ & \{\{P(\theta^{(1)} > 0|\overline{\mathbf{Y}_1}) < \epsilon_1\} \cap \{P(\theta^{(2)} > 0|\overline{\mathbf{Y}_1}) < \epsilon_1\}\} \\
& \cup \{\{P(\theta^{(1)} > 0|\overline{\mathbf{Y}_1}) < \epsilon_1\} \cap \{\epsilon_1 \leq P(\theta^{(2)} > 0|\overline{\mathbf{Y}_1}) < \eta_1\} \cap \{P(\theta^{(2)} > 0|\overline{\mathbf{Y}_2}) < \epsilon_2\}\} \\
& \cup \{\{P(\theta^{(2)} > 0|\overline{\mathbf{Y}_1}) < \epsilon_1\} \cap \{\epsilon_1 \leq P(\theta^{(1)} > 0|\overline{\mathbf{Y}_1}) < \eta_1\} \cap \{P(\theta^{(1)} > 0|\overline{\mathbf{Y}_2}) < \epsilon_2\}\} \\
& \cup \{\{\epsilon_1 \leq P(\theta^{(2)} > 0|\overline{\mathbf{Y}_1}) < \eta_1\} \cap \{P(\theta^{(2)} > 0|\overline{\mathbf{Y}_2}) < \epsilon_2\} \cap \{\epsilon_1 \leq P(\theta^{(1)} > 0|\overline{\mathbf{Y}_1}) < \eta_1\} \cap \{P(\theta^{(1)} > 0|\overline{\mathbf{Y}_2}) < \epsilon_2\}\}\Big\}
\end{aligned}
$$

The probability of R_2 is the sum of the probabilities of the four events in R_2. For the first event, we need to compute

$$P(\{P(\theta^{(1)} > 0|\overline{\boldsymbol{Y}_1}) < \epsilon_1\} \cap \{P(\theta^{(2)} > 0|\overline{\boldsymbol{Y}_1}) < \epsilon_1\}) = F_2\left(-\gamma_1^{(1)} + \frac{\mu_0^{(0)}\tau_{00}}{\tau_{00} + I_1^{(0)}} + z_{\epsilon_1}v_1^1, -\gamma_1^{(2)} + \frac{\mu_0^{(0)}\tau_{00}}{\tau_{00} + I_1^{(0)}} + z_{\epsilon_1}v_1^2\right),$$

where $F_2(x, y)$ is the cumulative density function of

$$\begin{pmatrix} \sum_{i=1}^2 c_{i1}^{(1)}\overline{Y}_1^{(i)} - c_1^{(0)}\overline{Y}_1^{(0)} \\ \sum_{i=1}^2 c_{i1}^{(2)}\overline{Y}_1^{(i)} - c_1^{(0)}\overline{Y}_1^{(0)} \end{pmatrix} \sim N\left[\begin{pmatrix} \sum_{i=1}^2 c_{i1}^{(1)}\mu^{(i)} - c_1^{(0)}\mu^{(0)} \\ \sum_{i=1}^2 c_{i1}^{(2)}\mu^{(i)} - c_1^{(0)}\mu^{(0)} \end{pmatrix}, \epsilon\right]$$

with

$$\epsilon = \begin{pmatrix} \sum_{i=1}^2 \frac{(c_{i1}^{(1)}\sigma_i)^2}{n_1^{(i)}} + \frac{(c_1^{(0)}\sigma_0)^2}{n_1^{(0)}} & \sum_{i=1}^2 c_{i1}^{(1)}c_{i1}^{(2)}\frac{\sigma_i^2}{n_1^{(i)}} + (c_1^{(0)})^2\frac{\sigma_0^2}{n_1^{(0)}} \\ \sum_{i=1}^2 c_{i1}^{(1)}c_{i1}^{(2)}\frac{\sigma_i^2}{n_1^{(i)}} + (c_1^{(0)})^2\frac{\sigma_0^2}{n_1^{(0)}} & \sum_{i=1}^2 \frac{(c_{i1}^{(2)}\sigma_i)^2}{n_1^{(i)}} + \frac{(c_1^{(0)}\sigma_0)^2}{n_1^{(0)}} \end{pmatrix}$$

$$c_{i1}^{(1)} = \left(\boldsymbol{\Gamma}_1^{-1}\boldsymbol{\Sigma}^{-1}\boldsymbol{n}_1\right)[1, i],\ \gamma_1^{(1)} = \left(\boldsymbol{\Gamma}_1^{-1}\boldsymbol{\Omega}^{-1}\boldsymbol{\mu}\right)[1], c_{i1}^{(2)} = \left(\boldsymbol{\Gamma}_1^{-1}\boldsymbol{\Sigma}^{-1}\boldsymbol{n}_1\right)[2, i], \gamma_1^{(2)} = \left(\boldsymbol{\Gamma}_1^{-1}\boldsymbol{\Omega}^{-1}\boldsymbol{\mu}\right)[2],\ \boldsymbol{n}_1 = (n_1^{(1)}, n_1^{(2)}).$$

Similarly, the probability of the second event of R_2 is

$$\begin{aligned} &P(\{P(\theta^{(1)} > 0|\overline{\boldsymbol{Y}_1}) < \epsilon_1\} \cap \{\epsilon_1 \le P(\theta^{(2)} > 0|\overline{\boldsymbol{Y}_1}) < \eta_1\} \cap \{P(\theta^{(2)} > 0|\overline{\boldsymbol{Y}_2}) < \epsilon_2\}) = \\ &F_3\left(-\gamma_1^{(1)} + \frac{\mu_0^{(0)}\tau_{00}}{\tau_{00} + I_1^{(0)}} + z_{\epsilon_1}v_1^1, -\gamma_1^{(2)} + \frac{\mu_0^{(0)}\tau_{00}}{\tau_{00} + I_1^{(0)}} + z_{\eta_1}v_1^2, -\gamma_2^{(2)} + \frac{\mu_0^{(0)}\tau_{00}}{\tau_{00} + I_2^{(0)}} + z_{\epsilon_2}v_2^2\right) - \\ &F_3\left(-\gamma_1^{(1)} + \frac{\mu_0^{(0)}\tau_{00}}{\tau_{00} + I_1^{(0)}} + z_{\epsilon_1}v_1^1, -\gamma_1^{(2)} + \frac{\mu_0^{(0)}\tau_{00}}{\tau_{00} + I_1^{(0)}} + z_{\epsilon_1}v_1^2, -\gamma_2^{(2)} + \frac{\mu_0^{(0)}\tau_{00}}{\tau_{00} + I_2^{(0)}} + z_{\epsilon_2}v_2^2\right), \end{aligned}$$

where $F_3(x, y, z)$ is the cumulative density function of

$$\begin{pmatrix} \sum_{i=1}^2 c_{i1}^{(1)}\overline{Y}_1^{(i)} - c_1^{(0)}\overline{Y}_1^{(0)} \\ \sum_{i=1}^2 c_{i1}^{(2)}\overline{Y}_1^{(i)} - c_1^{(0)}\overline{Y}_1^{(0)} \\ \sum_{i=1}^2 c_{i2}^{(2)}\overline{Y}_2^{(i)} - c_2^{(0)}\overline{Y}_2^{(0)} \end{pmatrix} \sim N\left[\begin{pmatrix} \sum_{i=1}^2 c_{i1}^{(1)}\mu^{(i)} - c_1^{(0)}\mu^{(0)} \\ \sum_{i=1}^2 c_{i1}^{(2)}\mu^{(i)} - c_1^{(0)}\mu^{(0)} \\ \sum_{i=1}^2 c_{i2}^{(2)}\mu^{(i)} - c_2^{(0)}\mu^{(0)} \end{pmatrix}, \epsilon\right]$$

with

$$\epsilon = \begin{pmatrix} \sum_{i=1}^2 \frac{(c_{i1}^{(1)}\sigma_i)^2}{n_1^{(i)}} + \frac{(c_1^{(0)}\sigma_0)^2}{n_1^{(0)}} & \sum_{i=1}^2 c_{i1}^{(1)}c_{i1}^{(2)}\frac{\sigma_i^2}{n_1^{(i)}} + (c_1^{(0)})^2\frac{\sigma_0^2}{n_1^{(0)}} & \omega_{13} \\ \sum_{i=1}^2 c_{i1}^{(1)}c_{i1}^{(2)}\frac{\sigma_i^2}{n_1^{(i)}} + (c_1^{(0)})^2\frac{\sigma_0^2}{n_1^{(0)}} & \sum_{i=1}^2 \frac{(c_{i1}^{(2)}\sigma_i)^2}{n_1^{(i)}} + \frac{(c_1^{(0)}\sigma_0)^2}{n_1^{(0)}} & \omega_{23} \\ \omega_{13} & \omega_{23} & \sum_{i=1}^2 \frac{(c_{i2}^{(2)}\sigma_i)^2}{n_2^{(i)}} + \frac{(c_2^{(0)}\sigma_0)^2}{n_2^{(0)}} \end{pmatrix}$$

$$c_{i2}^{(2)} = \left(\boldsymbol{\Gamma}_2^{-1}\boldsymbol{\Sigma}^{-1}\boldsymbol{n}_2\right)[2, i],\ \gamma_2^{(2)} = \left(\boldsymbol{\Gamma}_2^{-1}\boldsymbol{\Omega}^{-1}\boldsymbol{\mu}\right)[2],\ \boldsymbol{n}_2 = (n_1^{(1)}, 2n_1^{(2)}),\ n_2^{(0)} = 2n_1^{(0)}$$

$$\omega_{13} = c_{11}^{(1)}c_{12}^{(2)}\frac{\sigma_1^2}{n_1^{(1)}} + c_{21}^{(1)}c_{22}^{(2)}\frac{\sigma_2^2}{2n_1^{(2)}} + c_1^{(0)}c_2^{(0)}\frac{\sigma_0^2}{2n_1^{(0)}}, \omega_{23} = c_{11}^{(2)}c_{12}^{(2)}\frac{\sigma_1^2}{n_1^{(1)}} + c_{21}^{(2)}c_{22}^{(2)}\frac{\sigma_2^2}{2n_1^{(2)}} + c_1^{(0)}c_2^{(0)}\frac{\sigma_0^2}{2n_1^{(0)}}$$

The probability of the third event of R_2 is

$$\begin{aligned} &P(\{\epsilon_1 \le P(\theta^{(1)} > 0|\overline{\boldsymbol{Y}_1}) < \eta_1\} \cap \{P(\theta^{(2)} > 0|\overline{\boldsymbol{Y}_1}) < \epsilon_1\} \cap \{P(\theta^{(1)} > 0|\overline{\boldsymbol{Y}_2}) < \epsilon_2\}) = \\ &F_3\left(-\gamma_1^{(1)} + \frac{\mu_0^{(0)}\tau_{00}}{\tau_{00} + I_1^{(0)}} + z_{\eta_1}v_1^1, -\gamma_1^{(2)} + \frac{\mu_0^{(0)}\tau_{00}}{\tau_{00} + I_1^{(0)}} + z_{\epsilon_1}v_1^2, -\gamma_2^{(1)} + \frac{\mu_0^{(0)}\tau_{00}}{\tau_{00} + I_2^{(0)}} + z_{\epsilon_2}v_2^1\right) - \\ &F_3\left(-\gamma_1^{(1)} + \frac{\mu_0^{(0)}\tau_{00}}{\tau_{00} + I_1^{(0)}} + z_{\epsilon_1}v_1^1, -\gamma_1^{(2)} + \frac{\mu_0^{(0)}\tau_{00}}{\tau_{00} + I_1^{(0)}} + z_{\epsilon_1}v_1^2, -\gamma_2^{(1)} + \frac{\mu_0^{(0)}\tau_{00}}{\tau_{00} + I_2^{(0)}} + z_{\epsilon_2}v_2^1\right), \end{aligned}$$

where $F_3(x, y, z)$ is the cumulative density function of

$$\begin{pmatrix} \sum_{i=1}^{2} c_{i1}^{(1)}\overline{Y}_1^{(i)} - c_1^{(0)}\overline{Y}_1^{(0)} \\ \sum_{i=1}^{2} c_{i1}^{(2)}\overline{Y}_1^{(i)} - c_1^{(0)}\overline{Y}_1^{(0)} \\ \sum_{i=1}^{2} c_{i2}^{(1)}\overline{Y}_2^{(i)} - c_2^{(0)}\overline{Y}_2^{(0)} \end{pmatrix} \sim N\left[\begin{pmatrix} \sum_{i=1}^{2} c_{i1}^{(1)}\mu^{(i)} - c_1^{(0)}\mu^{(0)} \\ \sum_{i=1}^{2} c_{i1}^{(2)}\mu^{(i)} - c_1^{(0)}\mu^{(0)} \\ \sum_{i=1}^{2} c_{i2}^{(1)}\mu^{(i)} - c_2^{(0)}\mu^{(0)} \end{pmatrix}, \epsilon\right]$$

with

$$\begin{pmatrix} \sum_{i=1}^{2} \frac{(c_{i1}^{(1)}\sigma_i)^2}{n_1^{(i)}} + \frac{(c_1^{(0)}\sigma_0)^2}{n_1^{(0)}} & \sum_{i=1}^{2} c_{i1}^{(1)}c_{i1}^{(2)}\frac{\sigma_i^2}{n_1^{(i)}} + (c_1^{(0)})^2\frac{\sigma_0^2}{n_1^{(0)}} & \omega_{13} \\ \sum_{i=1}^{2} c_{i1}^{(1)}c_{i1}^{(2)}\frac{\sigma_i^2}{n_1^{(i)}} + (c_1^{(0)})^2\frac{\sigma_0^2}{n_1^{(0)}} & \sum_{i=1}^{2} \frac{(c_{i1}^{(2)}\sigma_i)^2}{n_1^{(i)}} + \frac{(c_1^{(0)}\sigma_0)^2}{n_1^{(0)}} & \omega_{23} \\ \omega_{13} & \omega_{23} & \sum_{i=1}^{2} \frac{(c_{i2}^{(1)}\sigma_i)^2}{n_2^{(i)}} + \frac{(c_2^{(0)}\sigma_0)^2}{n_2^{(0)}} \end{pmatrix}$$

$$c_{i2}^{(1)} = \left(\boldsymbol{\Gamma_2^{-1}\Sigma^{-1}n_2}\right)[1,i],\ \gamma_2^{(1)} = \left(\boldsymbol{\Gamma_2^{-1}\Omega^{-1}\mu}\right)[1],\ \boldsymbol{n_2} = (2n_1^{(1)}, n_1^{(2)}),\ n_2^{(0)} = 2n_1^{(0)}$$

$$\omega_{13} = c_{11}^{(1)}c_{12}^{(1)}\frac{\sigma_1^2}{2n_1^{(1)}} + c_{21}^{(1)}c_{22}^{(1)}\frac{\sigma_2^2}{n_1^{(2)}} + c_1^{(0)}c_2^{(0)}\frac{\sigma_0^2}{2n_1^{(0)}}, \omega_{23} = c_{11}^{(2)}c_{12}^{(1)}\frac{\sigma_1^2}{2n_1^{(1)}} + c_{21}^{(2)}c_{22}^{(1)}\frac{\sigma_2^2}{n_1^{(2)}} + c_1^{(0)}c_2^{(0)}\frac{\sigma_0^2}{2n_1^{(0)}}$$

Finally, the probability of the forth event of R_2 is

$$P(\{\epsilon_1 \le P(\theta^{(1)} > 0|\overline{\boldsymbol{Y_1}}) < \eta_1\} \cap \{\epsilon_1 \le P(\theta^{(2)} > 0|\overline{\boldsymbol{Y_1}}) < \eta_1\} \cap \{P(\theta^{(1)} > 0|\overline{\boldsymbol{Y_2}}) < \epsilon_2\} \cap \{P(\theta^{(2)} > 0|\overline{\boldsymbol{Y_2}}) < \epsilon_2\}) =$$

$$\int_{-\gamma_1^{(1)}+\frac{\mu_0^{(0)}\tau_{00}}{\tau_{00}+I_1^{(0)}}+z_{\epsilon_1}v_1^1}^{-\gamma_1^{(1)}+\frac{\mu_0^{(0)}\tau_{00}}{\tau_{00}+I_1^{(0)}}+z_{\eta_1}v_1^1} \int_{-\gamma_1^{(2)}+\frac{\mu_0^{(0)}\tau_{00}}{\tau_{00}+I_1^{(0)}}+z_{\epsilon_1}v_1^2}^{-\gamma_1^{(2)}+\frac{\mu_0^{(0)}\tau_{00}}{\tau_{00}+I_1^{(0)}}+z_{\eta_1}v_1^2} \int_{-\infty}^{-\gamma_2^{(1)}+\frac{\mu_0^{(0)}\tau_{00}}{\tau_{00}+I_2^{(0)}}+z_{\epsilon_2}v_2^1} \int_{-\infty}^{-\gamma_2^{(2)}+\frac{\mu_0^{(0)}\tau_{00}}{\tau_{00}+I_2^{(0)}}+z_{\epsilon_2}v_2^2} f(x,y,z,t)\,dxdydzdt$$

where $f(x, y, z, t)$ is the density function of

$$\begin{pmatrix} \sum_{i=1}^{2} c_{i1}^{(1)}\overline{Y}_1^{(i)} - c_1^{(0)}\overline{Y}_1^{(0)} \\ \sum_{i=1}^{2} c_{i1}^{(2)}\overline{Y}_1^{(i)} - c_1^{(0)}\overline{Y}_1^{(0)} \\ \sum_{i=1}^{2} c_{i2}^{(1)}\overline{Y}_2^{(i)} - c_2^{(0)}\overline{Y}_2^{(0)} \\ \sum_{i=1}^{2} c_{i2}^{(2)}\overline{Y}_2^{(i)} - c_2^{(0)}\overline{Y}_2^{(0)} \end{pmatrix} \sim N\left[\begin{pmatrix} \sum_{i=1}^{2} c_{i1}^{(1)}\mu^{(i)} - c_1^{(0)}\mu^{(0)} \\ \sum_{i=1}^{2} c_{i1}^{(2)}\mu^{(i)} - c_1^{(0)}\mu^{(0)} \\ \sum_{i=1}^{2} c_{i2}^{(1)}\mu^{(i)} - c_2^{(0)}\mu^{(0)} \\ \sum_{i=1}^{2} c_{i2}^{(2)}\mu^{(i)} - c_2^{(0)}\mu^{(0)} \end{pmatrix}, \epsilon\right]$$

with $\epsilon =$

$$\begin{pmatrix} \sum_{i=1}^{2} \frac{(c_{i1}^{(1)}\sigma_i)^2}{n_1^{(i)}} + \frac{(c_1^{(0)}\sigma_0)^2}{n_1^{(0)}} & \sum_{i=1}^{2} c_{i1}^{(1)}c_{i1}^{(2)}\frac{\sigma_i^2}{n_1^{(i)}} + (c_1^{(0)})^2\frac{\sigma_0^2}{n_1^{(0)}} & \omega_{13} & \omega_{14} \\ \sum_{i=1}^{2} c_{i1}^{(1)}c_{i1}^{(2)}\frac{\sigma_i^2}{n_1^{(i)}} + (c_1^{(0)})^2\frac{\sigma_0^2}{n_1^{(0)}} & \sum_{i=1}^{2} \frac{(c_{i1}^{(2)}\sigma_i)^2}{n_1^{(i)}} + \frac{(c_1^{(0)}\sigma_0)^2}{n_1^{(0)}} & \omega_{23} & \omega_{24} \\ \omega_{13} & \omega_{23} & \sum_{i=1}^{2} \frac{(c_{i2}^{(1)}\sigma_i)^2}{n_2^{(i)}} + \frac{(c_2^{(0)}\sigma_0)^2}{n_2^{(0)}} & \omega_{34} \\ \omega_{14} & \omega_{24} & \omega_{34} & \sum_{i=1}^{2} \frac{(c_{i2}^{(2)}\sigma_i)^2}{n_2^{(i)}} + \frac{(c_2^{(0)}\sigma_0)^2}{n_2^{(0)}} \end{pmatrix}$$

$$c_{i2}^{(1)} = \left(\boldsymbol{\Gamma_2^{-1}\Sigma^{-1}n_2}\right)[1,i],\ \gamma_2^{(1)} = \left(\boldsymbol{\Gamma_2^{-1}\Omega^{-1}\mu}\right)[1] + \frac{\mu_0^{(0)}\tau_{00}}{\tau_{00} + I_2^{(0)}},$$

$$c_{i2}^{(2)} = \left(\boldsymbol{\Gamma_2^{-1}\Sigma^{-1}n_2}\right)[2,i],\ \gamma_2^{(2)} = \left(\boldsymbol{\Gamma_2^{-1}\Omega^{-1}\mu}\right)[2],\ \boldsymbol{n_2} = (2n_1^{(1)}, 2n_1^{(2)}),\ n_2^{(0)} = 2n_1^{(0)}$$

$$\omega_{13} = c_{11}^{(1)}c_{12}^{(1)}\frac{\sigma_1^2}{2n_1^{(1)}} + c_{21}^{(1)}c_{22}^{(1)}\frac{\sigma_2^2}{2n_1^{(2)}} + c_1^{(0)}c_2^{(0)}\frac{\sigma_0^2}{2n_1^{(0)}}, \omega_{23} = c_{11}^{(2)}c_{12}^{(1)}\frac{\sigma_1^2}{2n_1^{(1)}} + c_{21}^{(2)}c_{22}^{(1)}\frac{\sigma_2^2}{2n_1^{(2)}} + c_1^{(0)}c_2^{(0)}\frac{\sigma_0^2}{2n_1^{(0)}}$$

$$\omega_{14} = c_{11}^{(1)}c_{12}^{(2)}\frac{\sigma_1^2}{2n_1^{(1)}} + c_{21}^{(1)}c_{22}^{(2)}\frac{\sigma_2^2}{2n_1^{(2)}} + c_1^{(0)}c_2^{(0)}\frac{\sigma_0^2}{2n_1^{(0)}}, \omega_{24} = c_{11}^{(2)}c_{12}^{(2)}\frac{\sigma_1^2}{2n_1^{(1)}} + c_{21}^{(2)}c_{22}^{(2)}\frac{\sigma_2^2}{2n_1^{(2)}} + c_1^{(0)}c_2^{(0)}\frac{\sigma_0^2}{2n_1^{(0)}}$$

$$\omega_{34} = c_{12}^{(1)}c_{12}^{(2)}\frac{\sigma_1^2}{2n_1^{(1)}} + c_{22}^{(1)}c_{22}^{(2)}\frac{\sigma_2^2}{2n_1^{(2)}} + c_2^{(0)}c_2^{(0)}\frac{\sigma_0^2}{2n_1^{(0)}}$$

2 | NUMERICAL RESULTS

The numerical results that compare the proposed Bayesian design with the frequentist designs with Pocock critical bounds are provided in Figures 1 and 2. The results under the global null hypothesis are provided in Table 1.

3 | FURTHER EXPLORATIONS OF THE OPERATING CHARACTERISTICS FOR A 4-ARM 2-STAGE B-MAMS DESIGN

In this section, further explorations of the probabilities to reject at least one null hypothesis for a 4-arm 2-stage design are provided. For the Bayesian model we set: $\tau = 340^{-2}, \tau_{01} = 10^{-6}, \mu_0^{(0)} = 489, \mu_0^{(1)} = 602, \delta_0^{(1)} = \delta_0^{(2)} = 0$. The precision values for the treatment mean differences and the control mean are given in Table 2. The Table provides also the sample sizes and the critical bounds that are found to control the FWER under the global and partial nulls at level $\alpha = 0.025$ when $\mu^{(0)} = 489$ and the design is powered at 80% to reject all hypotheses under $\boldsymbol{\theta} = (120, 120, 120)$.

It can be observed in Figures 3 and 4 that the B-MAMS(U) design is robust in controlling the FWER around the global and partial null hypotheses for all the considered values of the control mean. Under the global null hypothesis the FWER is equal to 0.025 when the control mean is $\mu^{(0)} = 489$ or $\mu^{(0)} = 1489$.

Regarding the B-MAMS(D) design, the results in Figures 5 and 6 show that the FWER is controlled under the global and partial null hypotheses when $\mu^{(0)} = 489$ and slightly inflated under the global null and the partial null - around 0.1% - when $\mu^{(0)} = 1489$. However, this design shows also some minor inflations - up to around 0.7% - when the treatment effects are close to 200.

The B-MAMS(C) design is robust in controlling the FWER when $\mu^{(0)} = 489$ - see Figure 7 - but it shows major inflations around the global and partial nulls when the control mean is off by a factor of 3 - see Figure 8. For example, for this chosen design, it can be observed that the probability to reject at least one hypothesis is almost 1 when $\theta^{(1)}$ or $\theta^{(2)}$ are equal to zero and $\theta^{(3)} = -200$.

Overall, as concluded for the 3-arm 2-stage design, these explorative analyses have shown minor inflations on the FWER when the true control mean is different from the prior assumption and the precision on the control mean is small. Thus, the proposed design can provide robust results when the assumption on the value of the control mean is violated. However, substantial inflations of the FWER are shown when an informative prior distribution is used for the control mean.

In addition, for the 4-arm 2-stage design, we have also included some results to compare how the chosen Bayesian designs perform in terms of rejecting all hypotheses and rejecting the third hypothesis - see Figures 9 and 10 respectively - compared to the frequentist ordered and multi-arm multi-stage designs under some alternative scenarios. Figure 11 shows the expected sample sizes (ESS) for the considered scenarios.

Overall, the results suggest that the proposed Bayesian design provides benefits compared to the considered frequentist approaches in terms of probability to reject the third hypothesis - an increase up to around 90% for the B-MAMS(D), B-MAMS(C) and B-MAMS(U) compared to the ORD - when $\theta^{(3)} = 120$ - see Figure 10. Nevertheless, the proposed design shows a reduction in power to reject all hypotheses - the B-MAMS(D) shows a difference up to around 5% compared to the ORD, while the B-MAMS(C) and B-MAMS(U) provide both a difference of around 5% compared to ORD designs respectively. In addition, when historical information is disregarded, the proposed design can match the operating characteristics of the MAMS(m) frequentist design. In terms of ESS, the B-MAMS(D) and B-MAMS(C) designs provide a reduction - up to 10% - compared to the frequentist MAMS(m) design.

4 | FURTHER EXPLORATIONS OF THE OPERATING CHARACTERISTICS FOR THE 3-ARM 3-STAGE B-MAMS DESIGN

Figure 12 provides further explorations about the probability to reject at least one null hypothesis when $\mu^{(0)} = 0, 0.5, 1$ for a 3-arm 3-stage B-MAMS design. For the Bayesian model we set: $\tau = 1, \tau_{01} = 10^{-6}, \mu_0^{(0)} = 0, \mu_0^{(1)} = 0, \delta_0^{(1)} = 0$. The precision values for the treatment mean differences and the control mean are given in Table 3. The Table provides also the sample sizes

and the critical bounds that are found to control the FWER under the global and partial nulls at level $\alpha = 0.025$ when $\mu^{(0)} = 0$ and the designs are powered at 80% to reject all hypotheses under $\boldsymbol{\theta} = (0.5, 0.5)$.

It can be observed that the design is robust in controlling the FWER around the global and null partial hypotheses for the considered values of the control mean for the B-MAMS(D) and B-MAMS(U) designs. The FWER is inflated for the B-MAMS(C) design when the control mean is different from $\mu^{(0)} = 0$.

ACKNOWLEDGMENTS

This report is independent research supported by the National Institute for Health Research (NIHR Advanced Fellowship, Dr Pavel Mozgunov, NIHR300576) and by the NIHR Cambridge Biomedical Research Centre (BRC-1215-20014). The views expressed in this publication are those of the authors and not necessarily those of the NHS, the National Institute for Health Research or the Department of Health and Social Care (DHSC). T Jaki and P Mozgunov also received funding from UK Medical Research Council (MC_UU_00002/14 and MC_UU_00002/19, respectively). For the purpose of open access, the author has applied a Creative Commons Attribution (CC BY) licence to any Author Accepted Manuscript version arising.

Author contributions

All authors have directly participated in the planning and execution of the presented work.

Financial disclosure

None reported.

Conflict of interest

The authors declare no potential conflict of interests.

How to cite this article:

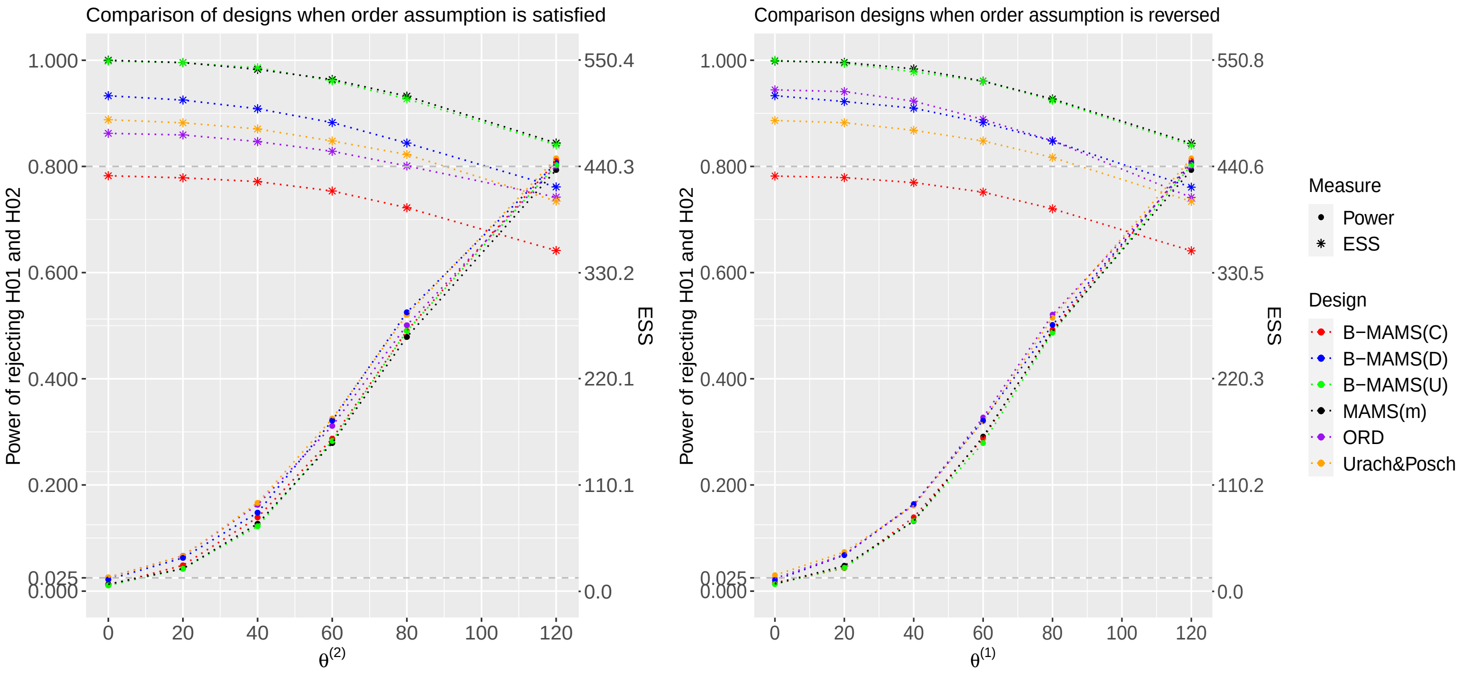

FIGURE 1 Power to reject all hypotheses and expected sample sizes (ESS) under $\boldsymbol{\theta} = (120, \theta^{(2)})$ and $\theta^{(2)} \in \{0, 20, 40, 60, 80, 120\}$ (left) and under $\boldsymbol{\theta} = (\theta^{(1)}, 120)$ and $\theta^{(1)} \in \{0, 20, 40, 60, 80, 120\}$ (right) for the 3-arm 2-stage MAMS(m), ORD, Urach&Posch and Bayesian designs when all designs are powered at 80% to reject both hypotheses under $\boldsymbol{\theta} = (120, 120)$. All designs use Pocock bounds. Results are provided using 5000 replications.

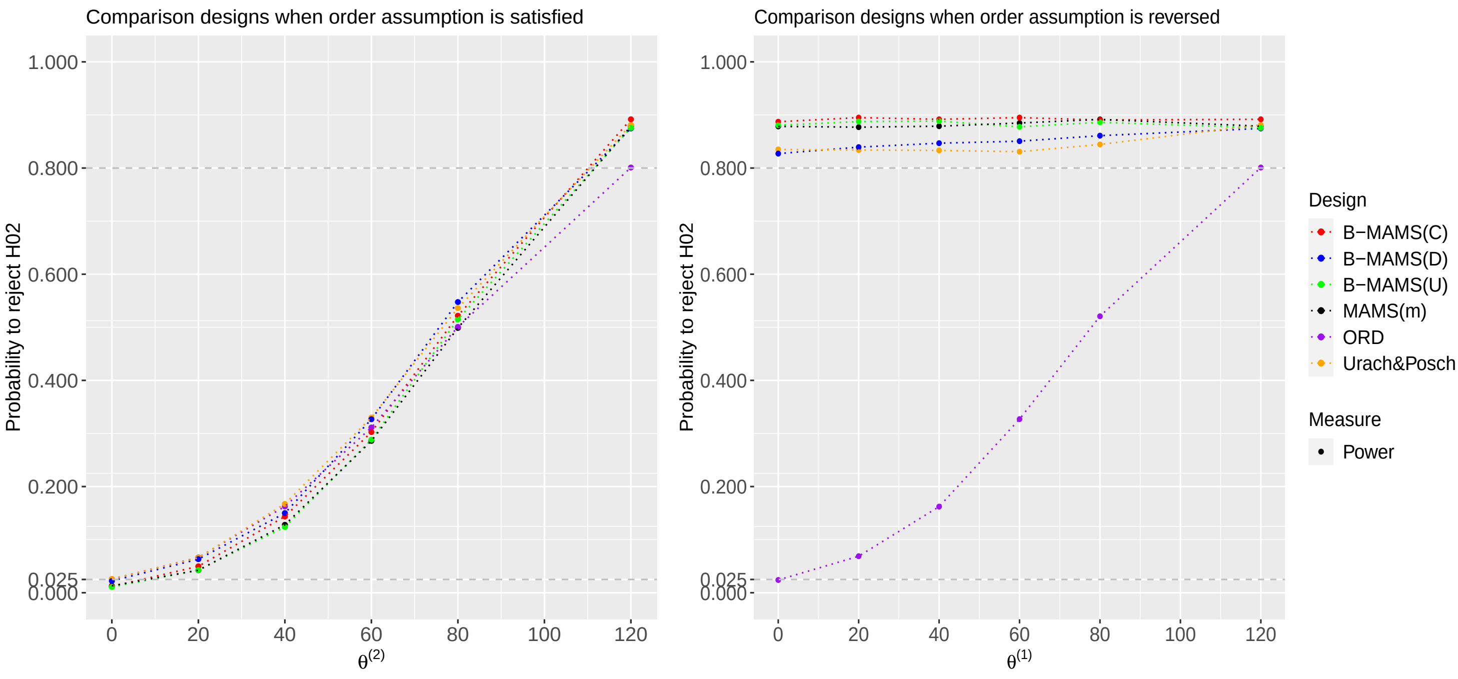

FIGURE 2 Probability to reject H_{02} under $\boldsymbol{\theta} = (120, \theta^{(2)})$ and $\theta^{(2)} \in \{0, 20, 40, 60, 80, 120\}$ (left) and under $\boldsymbol{\theta} = (\theta^{(1)}, 120)$ and $\theta^{(1)} \in \{0, 20, 40, 60, 80, 120\}$ (right) for for the 3-arm 2-stage MAMS(m), ORD, Urach&Posch and Bayesian designs when all designs are powered at 80% to reject both hypotheses under $\boldsymbol{\theta} = (120, 120)$. All designs use Pocock bounds. Results are provided using 5000 replications.

TABLE 1 Probability to reject at least one hypothesis (FWER), maximum sample size (Max SS) and expected sample size (ESS) for each design under the global null hypothesis H_0 with $\mu^{(1)} = \mu^{(2)} = \mu^{(0)} = 489$ when all designs are powered at 80% to reject all hypotheses under $\boldsymbol{\theta} = (120, 120)$. Results are provided using 5000 simulations. Upper - $u_j, j \in \{1, 2\}$ - and lower - l_1 - Pocock critical bounds are provided for the ORD and MAMS(m) frequentist designs. For the Urach and Posch design $u_j, j \in \{1, 2\}$ and l_1 are the upper and lower global boundaries, while $v_j, j \in \{1, 2\}$ are the elementary boundaries.

Design	u_1, u_2, l_1, v_1, v_2	$\eta_1, \eta_2, \epsilon_1$	Max SS	ESS	FWER
MAMS(m)	2.422, 2.422, -2.422, -, -		606	602.71	0.025
B-MAMS(U)		0.9923, 0.9923, 0.0077	606	602.80	0.025
ORD	2.179, 2.179, -2.179, -, -		528	521.22	0.025
B-MAMS(D)		0.9911, 0.9911, 0.089	558	554.73	0.025
Urach&Posch	2.422, 2.422, -2.422, 2.179, 2.179		534	530.65	0.027
B-MAMS(C)		0.9886, 0.9886, 0.0114	480	477.65	0.026

TABLE 2 Maximum sample size (Max SS), critical boundaries, prior information on the mean treatment differences and on the control mean for each design under the global null hypothesis H_0 with $\mu^{(1)} = \mu^{(2)} = \mu^{(3)} = \mu^{(0)} = 489$ when all designs are powered at 80% to reject all hypotheses under $\boldsymbol{\theta} = (120, 120, 120)$.

Design	u_1, u_2, l_1	$\eta_1, \eta_2, \epsilon_1$	Max SS	$\tau_d^{(1)}$	$\tau_d^{(2)}$	τ_{00}
B-MAMS(U)		0.9957, 0.9933, 0.8094	968	10^{-6}	10^{-6}	10^{-6}
B-MAMS(D)		0.9950, 0.9924, 0.8047	904	5.9×10^{-5}	5.9×10^{-5}	10^{-6}
B-MAMS(C)		0.9946, 0.9918, 0.8022	872	10^{-6}	10^{-6}	2.14×10^{-4}
MAMS(m)	2.6251, 2.4750, 0.8750		968			
ORD	2.2230, 2.0958, 0.7410		792			

TABLE 3 Maximum sample size (Max SS), critical boundaries, prior information on the mean treatment differences and on the control mean for each design under the global null hypothesis H_0 with $\mu^{(1)} = \mu^{(2)} = \mu^{(0)} = 0$ when all designs are powered at 80% to reject all hypotheses under $\boldsymbol{\theta} = (.5, .5)$.

Design	$\eta_1, \eta_2, \eta_3, \epsilon_1, \epsilon_2$	Max SS	$\tau_d^{(1)}$	τ_{00}
B-MAMS(U)	0.9969, 0.9922, 0.9911, 0.5, 0.9266	324	10^{-6}	10^{-6}
B-MAMS(D)	0.9966, 0.9916, 0.9904, 0.5, 0.9244	306	2	10^{-6}
B-MAMS(C)	0.9956, 0.9897, 0.9883, 0.5, 0.9176	261	10^{-6}	15

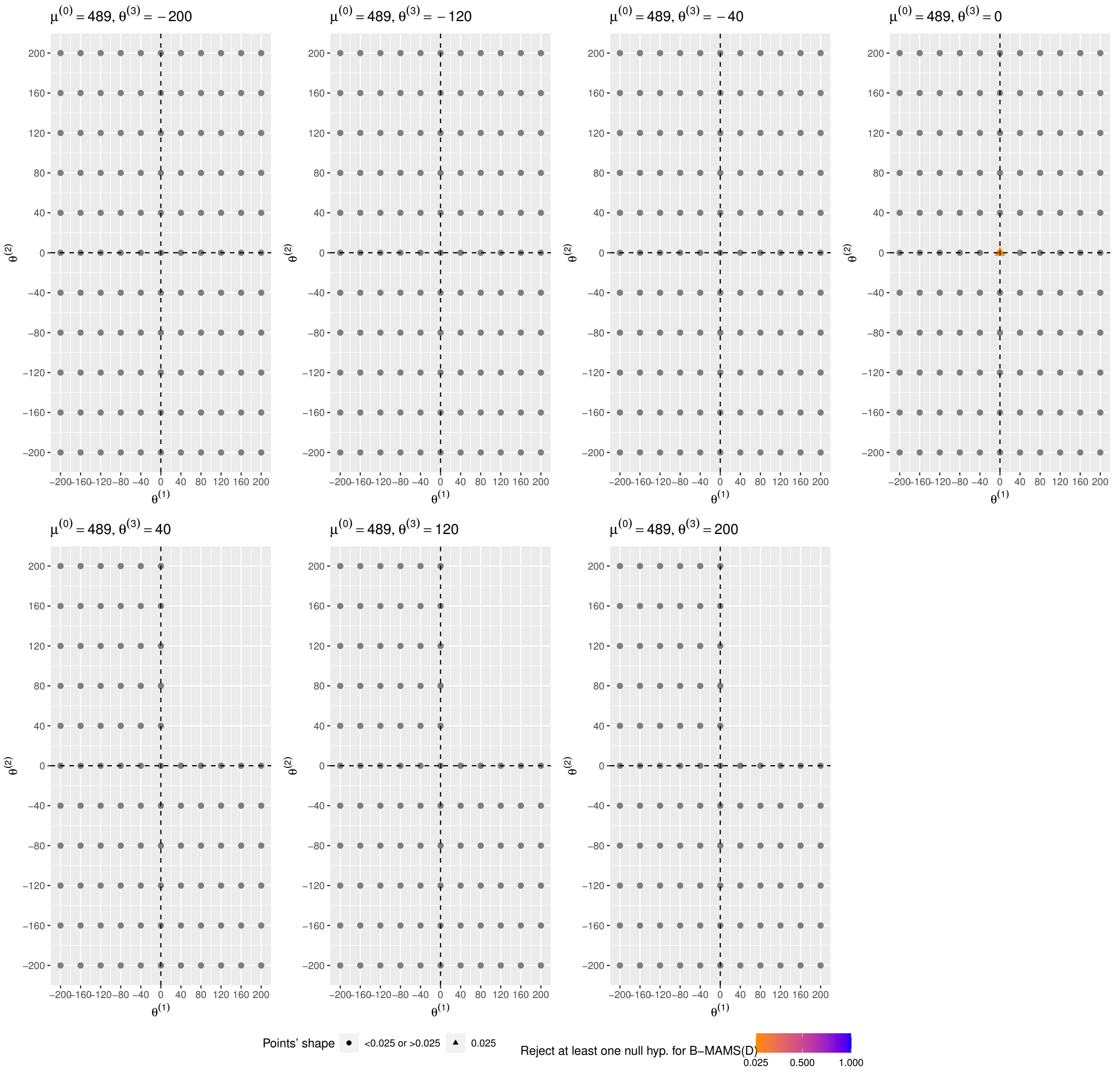

FIGURE 3 Probability to reject at least one null hypothesis when $\mu^{(0)} = 489$ for different values of the treatment effects for the 4-arm 2-stage B-MAMS(U) design when it is powered at 80% to reject both hypotheses under $\boldsymbol{\theta} = (120, 120, 120)$.

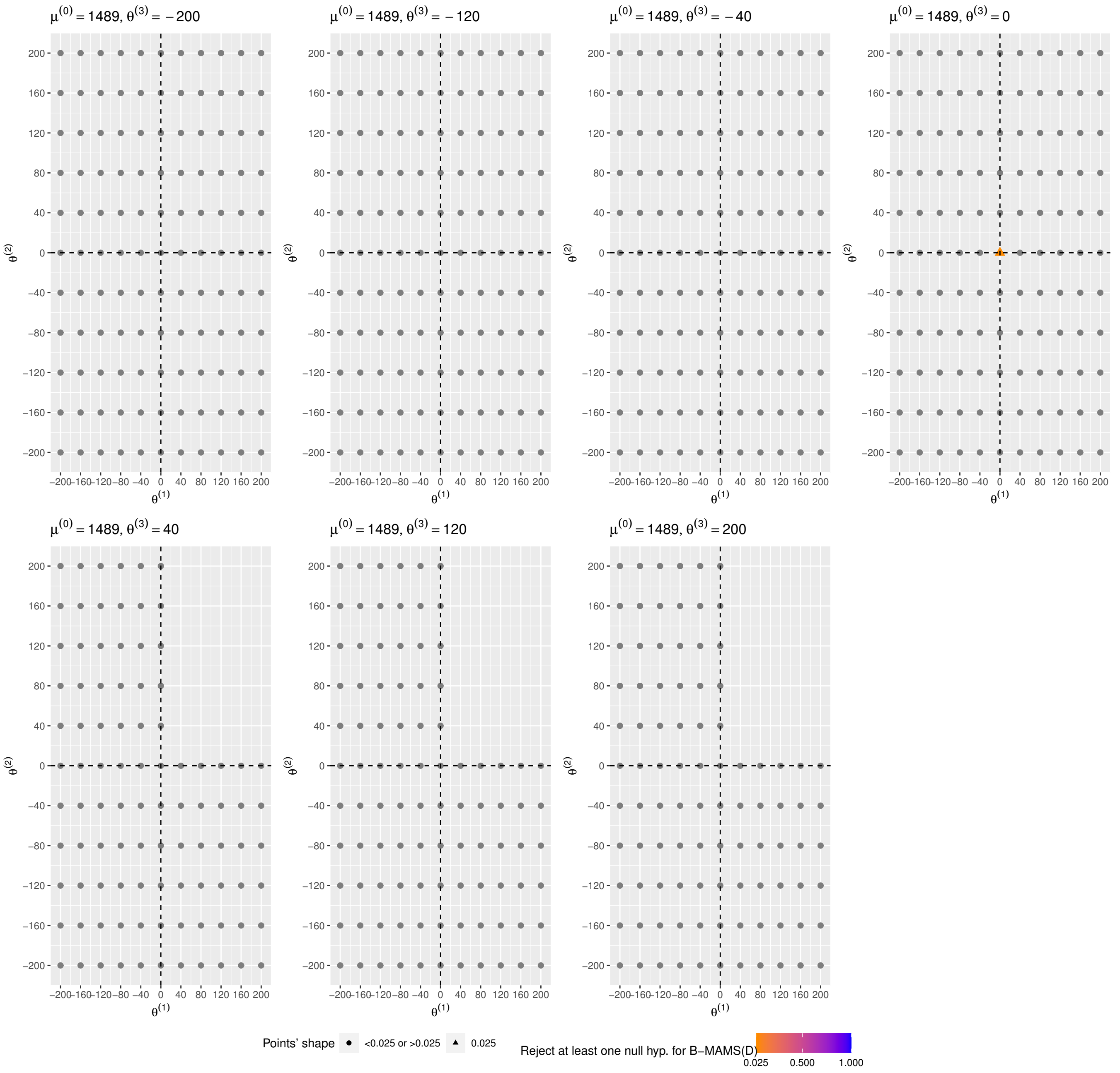

FIGURE 4 Probability to reject at least one null hypothesis when $\mu^{(0)} = 1489$ for different values of the treatment effects for the 4-arm 2-stage B-MAMS(U) design when it is powered at 80% to reject both hypotheses under $\boldsymbol{\theta} = (120, 120, 120)$.

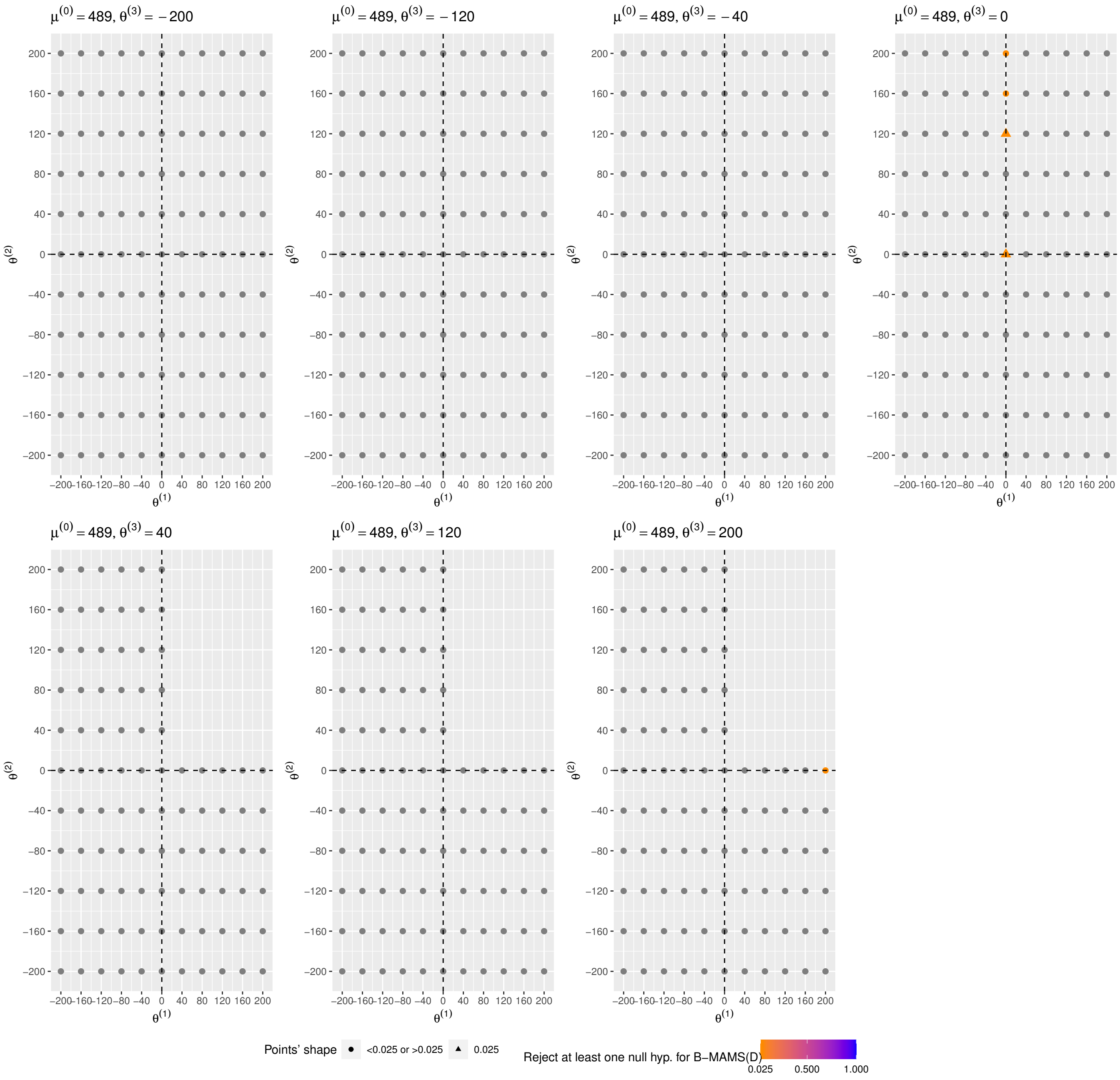

FIGURE 5 Probability to reject at least one null hypothesis when $\mu^{(0)} = 489$ for different values of the treatment effects for the 4-arm 2-stage B-MAMS(D) design when it is powered at 80% to reject both hypotheses under $\boldsymbol{\theta} = (120, 120, 120)$.

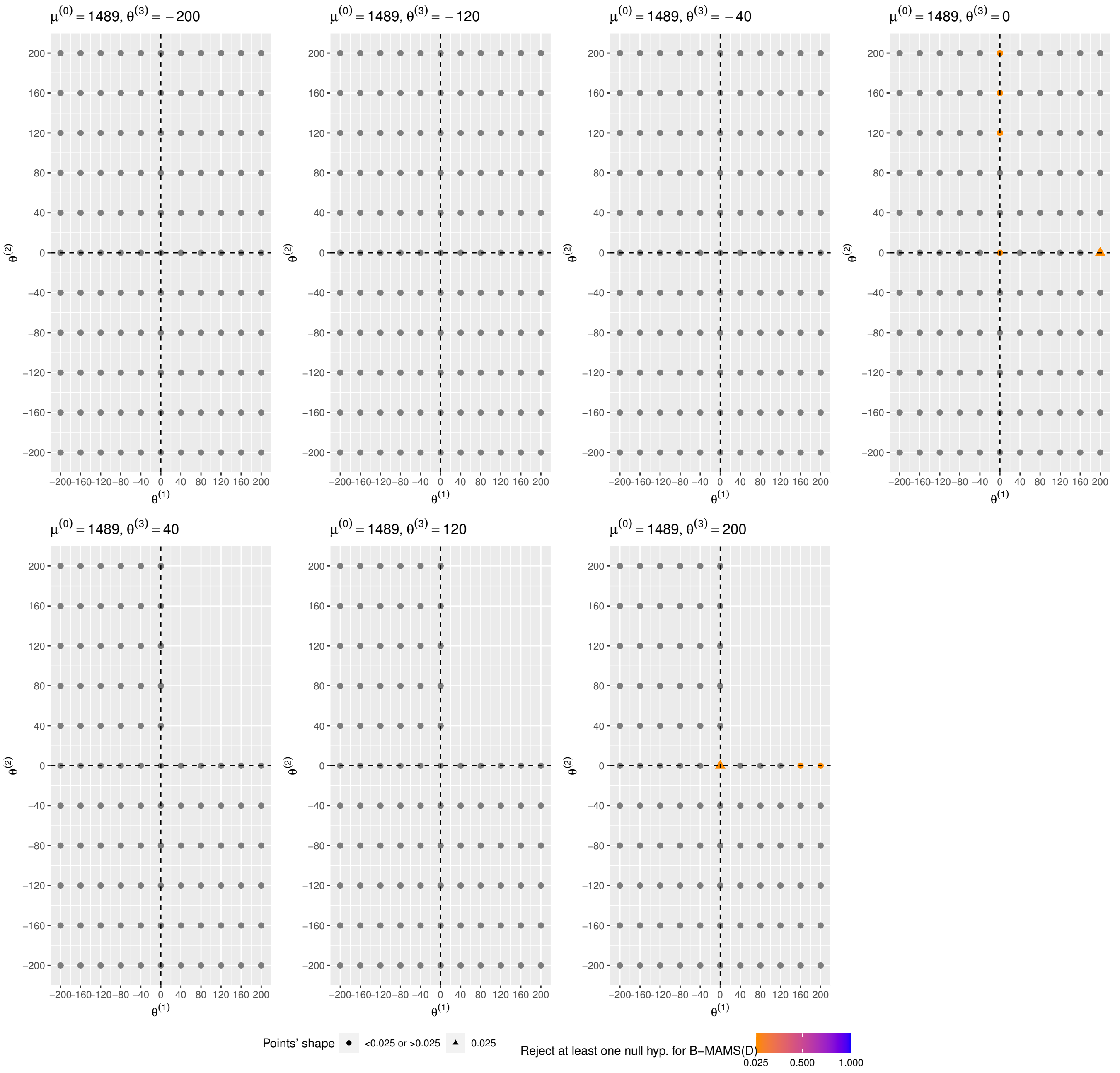

FIGURE 6 Probability to reject at least one null hypothesis when $\mu^{(0)} = 1489$ for different values of the treatment effects for the 4-arm 2-stage B-MAMS(D) design when it is powered at 80% to reject both hypotheses under $\boldsymbol{\theta} = (120, 120, 120)$.

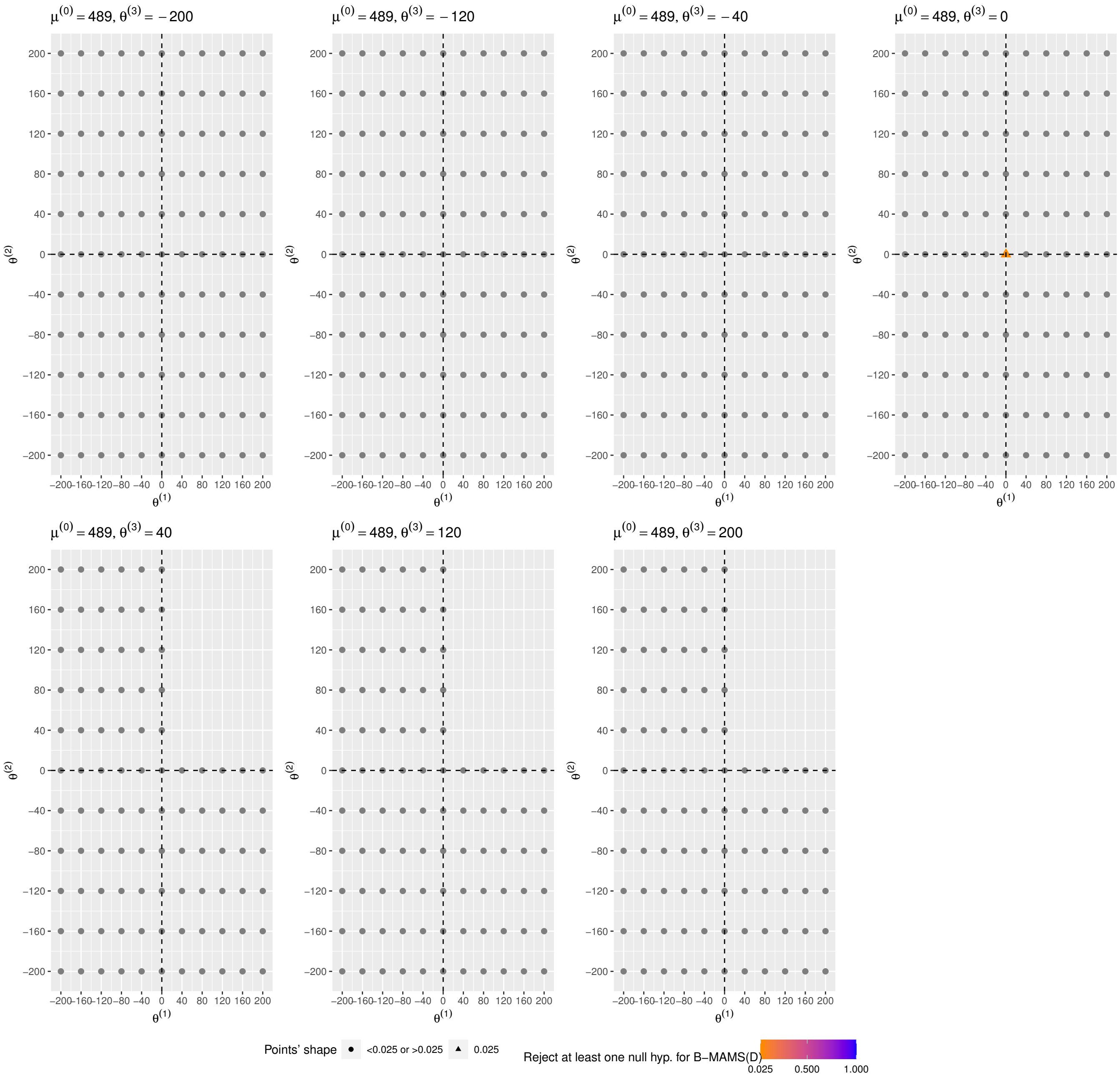

FIGURE 7 Probability to reject at least one null hypothesis when $\mu^{(0)} = 489$ for different values of the treatment effects for the 4-arm 2-stage B-MAMS(C) design when it is powered at 80% to reject both hypotheses under $\boldsymbol{\theta} = (120, 120, 120)$.

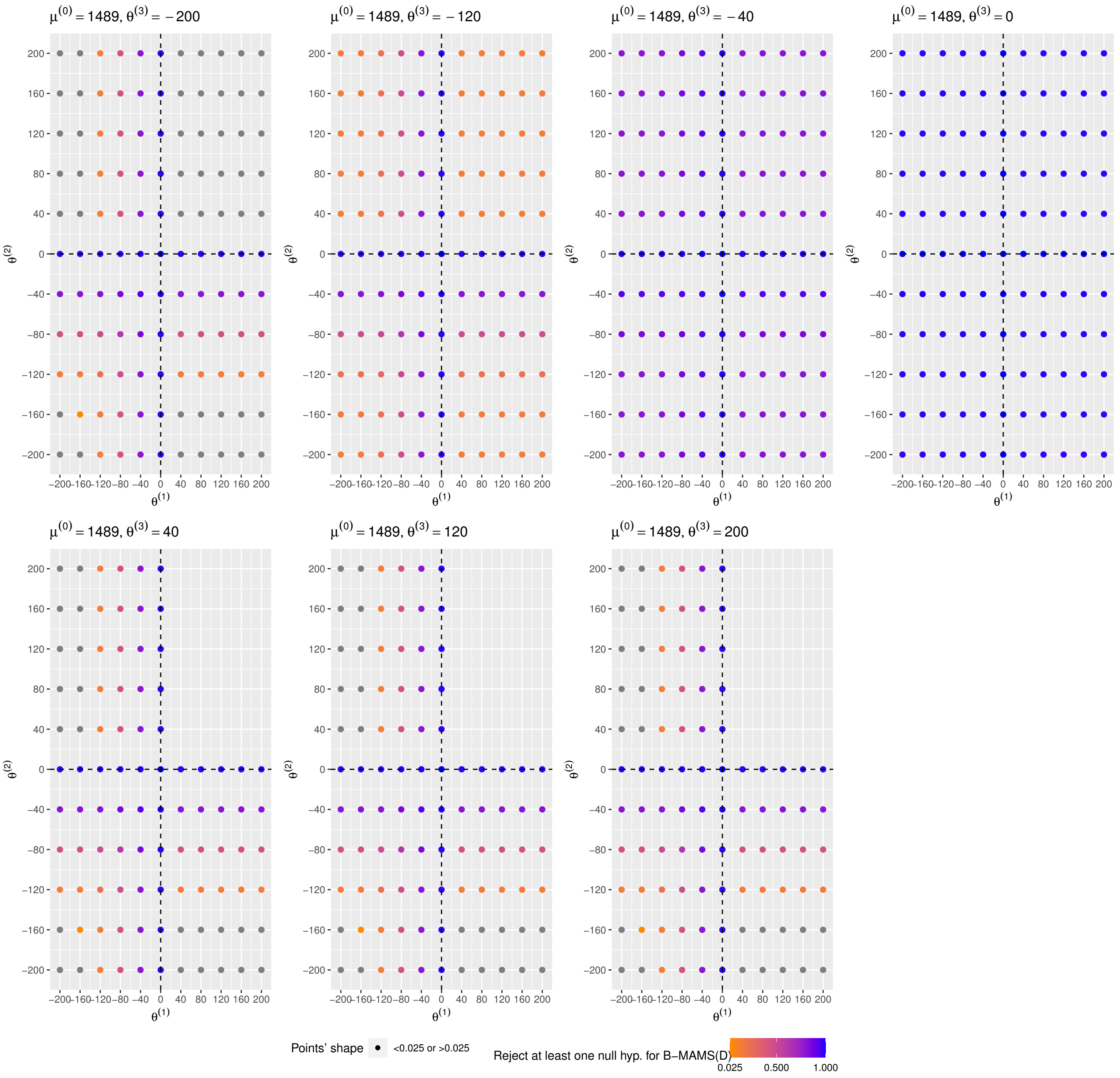

FIGURE 8 Probability to reject at least one null hypothesis when $\mu^{(0)} = 1489$ for different values of the treatment effects for the 4-arm 2-stage B-MAMS(C) design when it is powered at 80% to reject both hypotheses under $\boldsymbol{\theta} = (120, 120, 120)$.

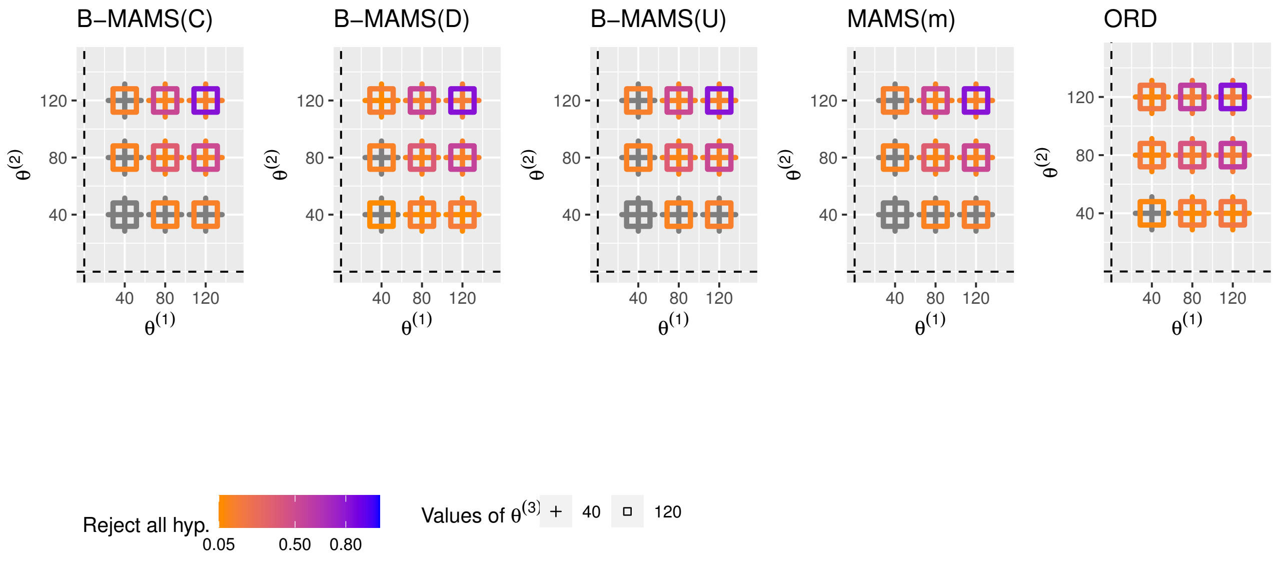

FIGURE 9 Probability to reject all hypotheses when $\mu^{(0)} = 489$ for different values of the treatment effects for the 4-arm 2-stage B-MAMS(U), B-MAMS(D), B-MAMS(C), MAMS(m), ORD designs when all are powered at 80% to reject all hypotheses under $\boldsymbol{\theta} = (120, 120, 120)$. All designs use triangular bounds. Results are provided using 10^4 replications.

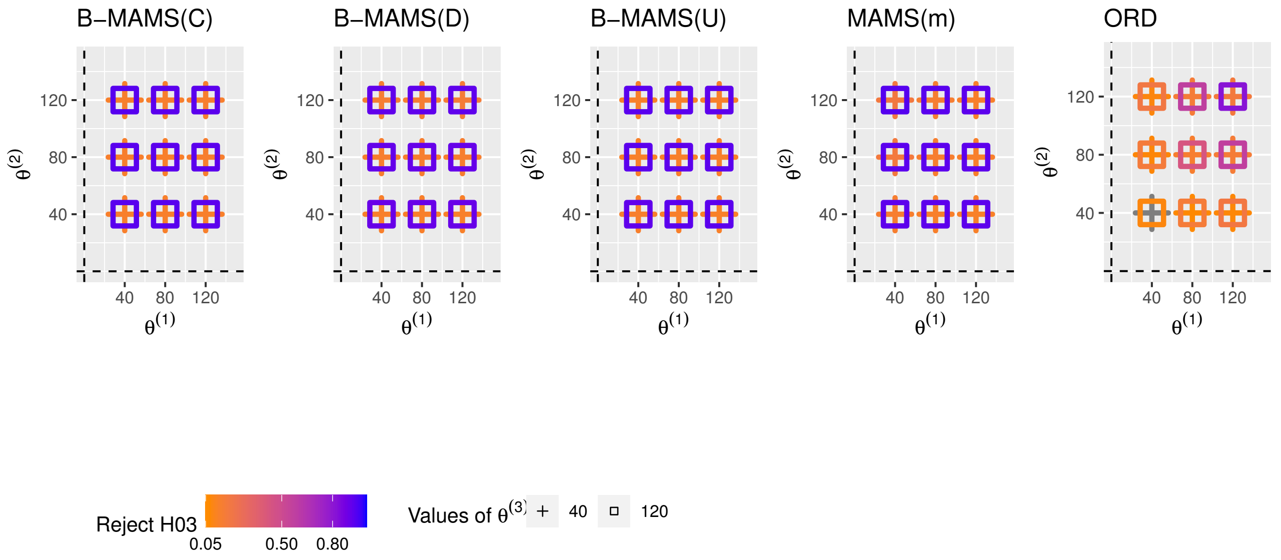

FIGURE 10 Probability to reject H_{03} when $\mu^{(0)} = 489$ for different values of the treatment effects for the 4-arm 2-stage B-MAMS(U), B-MAMS(D), B-MAMS(C), MAMS(m), ORD designs when all are powered at 80% to reject all hypotheses under $\boldsymbol{\theta} = (120, 120, 120)$. All designs use triangular bounds. Results are provided using 10^4 replications.

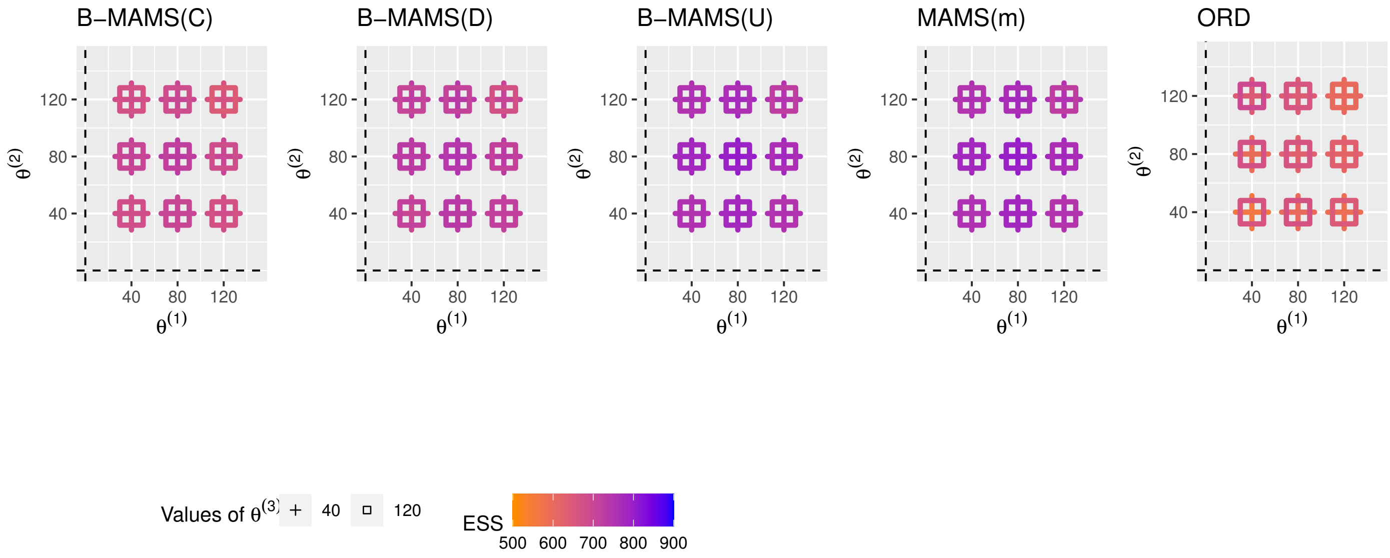

FIGURE 11 Expected sample sizes (ESS) when $\mu^{(0)} = 489$ for different values of the treatment effects for the 4-arm 2-stage B-MAMS(U), B-MAMS(D), B-MAMS(C), MAMS(m), ORD designs when all are powered at 80% to reject all hypotheses under $\boldsymbol{\theta} = (120, 120, 120)$. All designs use triangular bounds. Results are provided using 10^4 replications.

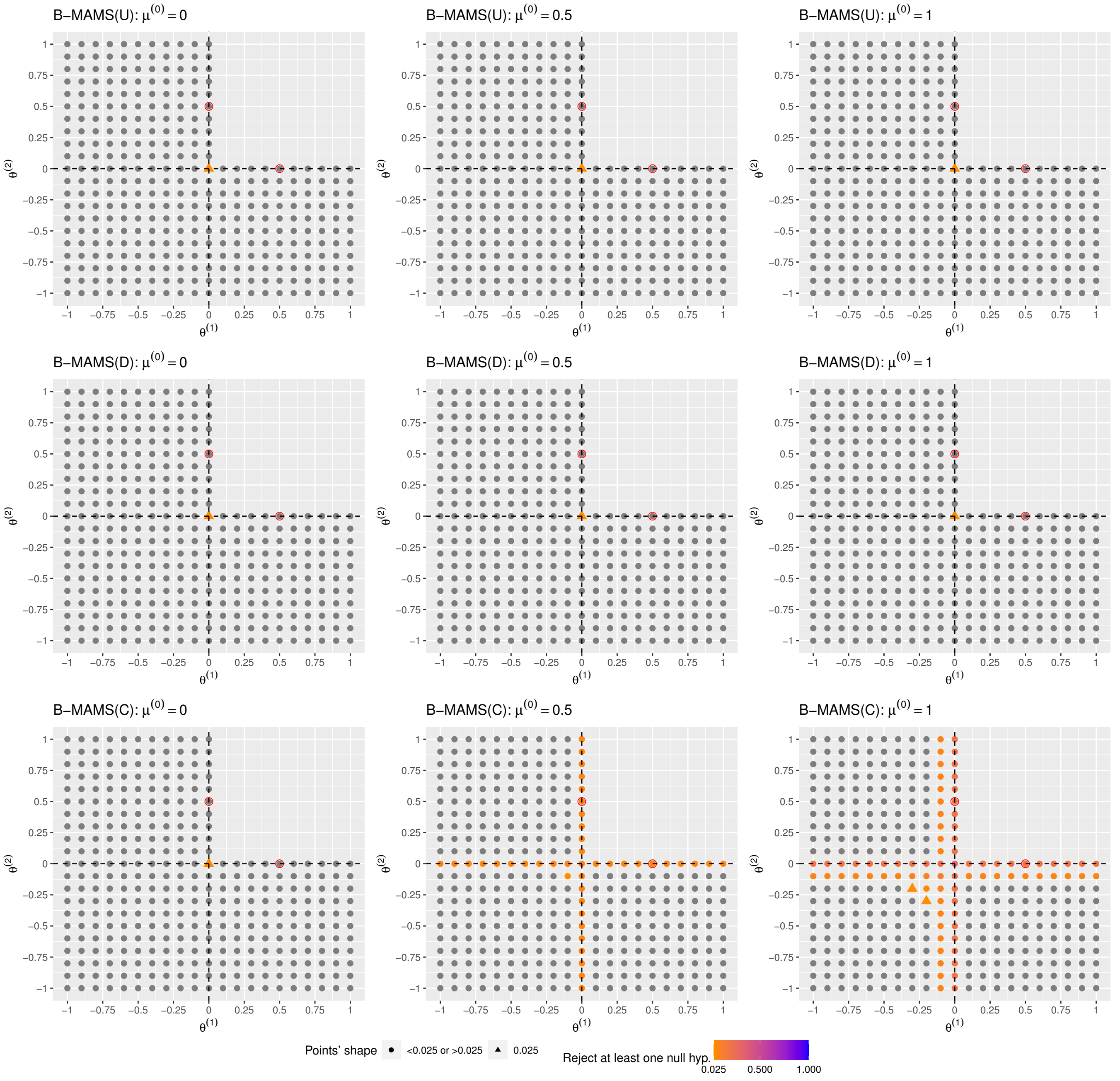

FIGURE 12 Probability to reject at least one null hypothesis when $\mu^{(0)} = 0, 0.5, 1$ for different values of the treatment effects for the 3-arm 3-stage B-MAMS(C), B-MAMS(D), B-MAMS(U) designs when they are powered at 80% to reject both hypotheses under $\boldsymbol{\theta} = (0.5, 0.5)$. Points in grey color refer to probability values below 0.025. Partial null configurations - $\boldsymbol{\theta} = (0.5, 0)$ and $\boldsymbol{\theta} = (0, 0.5)$ - are marked with red circles.
